# Mitochondria‐Targeted Nanoadjuvants Induced Multi‐Functional Immune‐Microenvironment Remodeling to Sensitize Tumor Radio‐Immunotherapy

**DOI:** 10.1002/advs.202400297

**Published:** 2024-05-05

**Authors:** Zaigang Zhou, Cheng Li, Chao Li, Lei Zhou, Shuo Tan, Weibin Hou, Congying Xie, Long Wang, Jianliang Shen, Wei Xiong

**Affiliations:** ^1^ Department of Urology The Third Xiangya Hospital of Central South University Changsha 410013 China; ^2^ National Engineering Research Center of Ophthalmology and Optometry Eye Hospital Wenzhou Medical University Wenzhou Zhejiang 325027 China; ^3^ Zhejiang Engineering Research Center for Tissue Repair Materials Wenzhou Institute University of Chinese Academy of Sciences Wenzhou Zhejiang 325001 China; ^4^ Zhejiang Engineering Research Center for Innovation and Application of Intelligent Radiotherapy Technology Zhejiang‐Hong Kong Precision Theranostics of Thoracic Tumors Joint Laboratory Wenzhou key Laboratory of Basic Science and Translational Research of Radiation Oncology The Second Affiliated Hospital of Wenzhou Medical University Wenzhou 325000 China

**Keywords:** collagen, immune‐microenvironment, mitochondria metabolism, nanoadjuvants, radiotherapy

## Abstract

It is newly revealed that collagen works as a physical barrier to tumor immune infiltration, oxygen perfusion, and immune depressor in solid tumors. Meanwhile, after radiotherapy (RT), the programmed death ligand‐1 (PD‐L1) overexpression and transforming growth factor‐β (TGF‐β) excessive secretion would accelerate DNA damage repair and trigger T cell exclusion to limit RT efficacy. However, existing drugs or nanoparticles can hardly address these obstacles of highly effective RT simultaneously, effectively, and easily. In this study, it is revealed that inducing mitochondria dysfunction by using oxidative phosphorylation inhibitors like Lonidamine (LND) can serve as a highly effective multi‐immune pathway regulation strategy through PD‐L1, collagen, and TGF‐β co‐depression. Then, IR‐LND is prepared by combining the mitochondria‐targeted molecule IR‐68 with LND, which then is loaded with liposomes (Lip) to create IR‐LND@Lip nanoadjuvants. By doing this, IR‐LND@Lip more effectively sensitizes RT by generating more DNA damage and transforming cold tumors into hot ones through immune activation by PD‐L1, collagen, and TGF‐β co‐inhibition. In conclusion, the combined treatment of RT and IR‐LND@Lip ultimately almost completely suppressed the growth of bladder tumors and breast tumors.

## Graphical abstract

In this research, IR‐LND@Lip is prepared by conjugating heptamethylene cyanine with Lonidamine to lower the dosage of Lonidamine needed in disrupting mitochondrial oxidative phosphorylation. By doing this, IR‐LND@Lip more effectively sensitizes radiotherapy by generating more DNA damage and transforming cold tumors into hot ones through hypoxia reversion and immune activation by PD‐L1, collagen, and TGF‐β co‐inhibition.

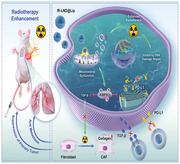

## 1 Introduction

As we all know, the aberrant high tumor interstitial pressure partly induced by the widely distributed collagen in the tumor was a main obstacle to effective tumor therapies by inducing tumor hypoxia and impairing drug accumulation, as well as causing T cell exhaustion via impairing T cell infiltration.^[^ ^1^ ^]^ Taking radiotherapy (RT) as an example, it has the capacity to generate significant amounts of reactive oxygen species (ROS) to induce DNA damage and induce DNA damage process when enough oxygen is supplied, which always is impaired due to the limited oxygen perfusion when too much collagen is located in solid tumors.^[^ ^2^ ^]^ Besides, RT can also induce immunogenic cell death (ICD) to recruit T cells to tumors by amplifying the release of high mobility group box 1 (HMGB1) and inducing the tumor membrane location of calreticulin (CRT), which then is also limited by collagen.^[^ ^3^ ^]^ Generally, collagen depletion via collagenase or small molecular collagen secretion direct or indirect inhibitor like pirfenidone was the most conventional method, but the lack of tumor‐targeting capacity, as well as its side effects, still led to the failure of these strategies.^[^ ^4^ ^]^ Moreover, these methods used to deplete collagen could hardly decrease or even increase the expression levels of programmed death ligand‐1 (PD‐L1), which still could not realize the most effective RT sensitization in solid tumors since PD‐L1 could not only work as an immune checkpoint inhibitor but also a DNA damage repair accelerator.^[^ ^5^ ^]^ Thus, more effective methods that could selectively and easily inhibit PD‐L1 and collagen simultaneously are still urgently needed.

Recently, it has been inconceivable that mitochondria metabolism chaos including oxidative phosphorylation (OXPHOS) remission plays a vital role in the immune exhaustion status in solid tumors.^[^ ^6^ ^]^ To this end, with the raised levels of OXPHOS, more adenosine triphosphate (ATP) was generated, which then could induce decreased adenosine diphosphate (ADP)/ATP or adenosine monophosphate (AMP)/ATP levels.^[^ ^7^ ^]^ Owing to this, the phosphorylation of AMP‐activated protein kinase (p‐AMPK) is decreased, which then could be reversed by OXPHOS remission.^[^ ^8^ ^]^ Generally, the expression levels of p‐AMPK are positively related to the amounts of tumor membrane and cytoplasm located PD‐L1 proteins since p‐AMPK promotes the phosphorylation of PD‐L1 at the S195 site to weaken the glycosylation of PD‐L1 protein with the following amplified degradation of PD‐L1 protein by proteasome.^[^ ^9^ ^]^ Additionally, the secretion of transforming growth factor‐β (TGF‐β) from tumor cells may also be inhibited by increased AMPK phosphorylation, thereby leading to immune activation and the subsequent potential reversal of fibroblast activation and remission of fibrosis development.^[^ ^10^ ^]^ Thus, OXPHOS may work as a highly effective PD‐L1 and TGF‐β depressor via inducing AMPK phosphorylation.^[^ ^9^, ^11^ ^]^ Apart from this, the activation of AMPK may impair the release or generation of collagen from cancer cells, resulting in immune system stimulation and enhanced oxygen perfusion.^[^ ^11^ ^]^ Hence, OXPHOS inhibition could be used as a highly effective adjuvant approach to sensitize RT by dual‐stage hypoxia reversion through enhanced oxygen perfusion and decreased oxygen consumption, as well as converting cold tumors to hot ones by enhancing T cell infiltration and its activity through collagen, PD‐L1, and TGF‐β co‐depression.^[^ ^6^, ^12^ ^]^ However, it remains to be confirmed whether this strategy can work as we expected since its potential has been largely overlooked.

**Scheme 1:**
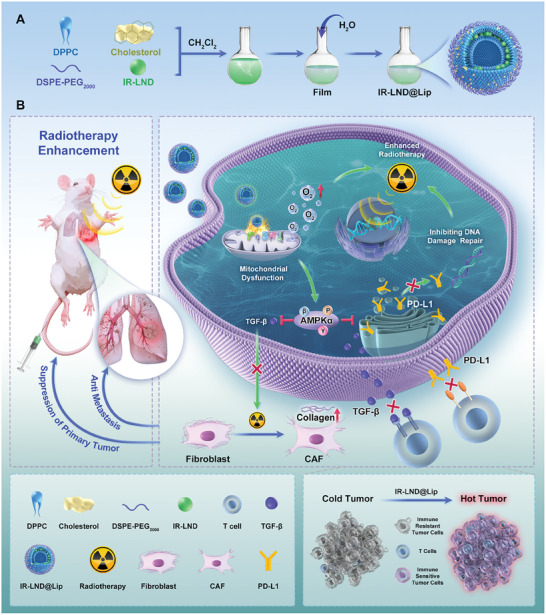
The schematic diagram of the synthetic route for IR‐LND@Lip nanoadjuvants and their mechanism for enhancing radio‐immunotherapy in the treatment of malignant tumors.

**Figure 1:**
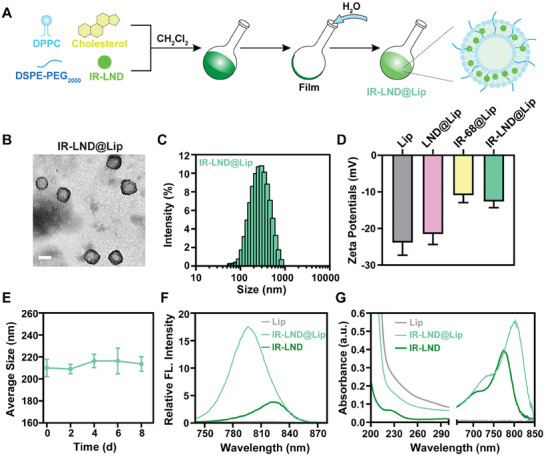
Characterization of the IR‐LND@Lip. A) The synthesis route of IR‐LND@Lip nanoadjuvants via a thin‐film hydration method. B) The TEM image of IR‐LND@Lip nanoparticles. Scale bars = 200 nm. C) Hydrodynamic diameters of IR‐LND@Lip nanoparticles. D) Zeta potentials of different liposomes (n = 3). E) Storage stability of IR‐LND@Lip nanoadjuvants in PBS at room temperature for 8 days (n = 3). F) Fluorescence spectra of Blank Lip, IR‐LND, and IR‐LND@Lip in deionized water. G) UV–vis spectra of Blank Lip, IR‐LND, and IR‐LND@Lip in deionized water.

**Figure 2:**
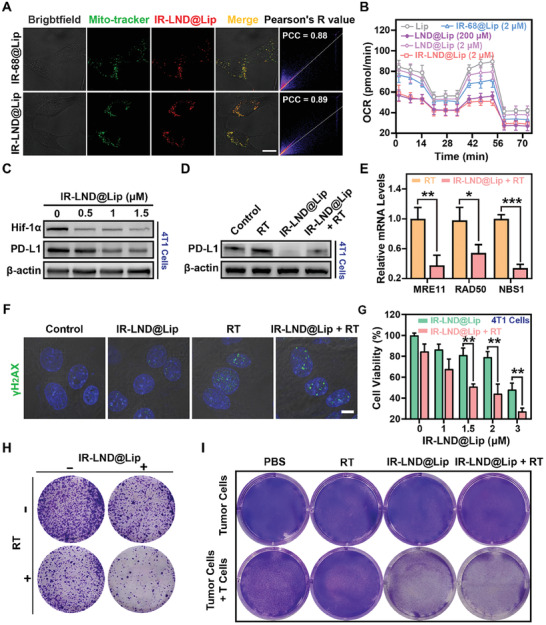
IR‐LND@Lip enhanced radio‐immunotherapy by reversing the tumor's hypoxic microenvironment and reducing PD‐L1 protein. A) Subcellular co‐location of IR‐68@Lip or IR‐LND@Lip with Mito‐Tracker Green in 4T1 cells, scale bar = 10 µm. B) The relative OCR of 4T1 cells received indicated treatments at different points in time (n = 3). C) Detection of the expression of HIF‐1α and PD‐L1 protein in 4T1 cells by western blotting after treatment with indicated doses of IR‐LND@Lip (n = 3). D) Detection of the expression of PD‐L1 protein in 4T1 cells by western blotting after different treatments (n = 3). E) Determination of mRNA levels of MRE11/RAD50/NBS1 in 4T1 cells by RT‐qPCR after different treatments (n = 3). F) Representative CLSM images of γ‐H2AX in 4T1 cells after indicated treatments, scale bar = 10 µm. G) Detection of the cell viability of 4T1 cells by CCK‐8 assay after different treatments with various concentrations of IR‐LND@Lip with or without RT co‐treatment (n = 6). H) The representative picture of the 4T1 cell clones after different treatments with IR‐LND@Lip (1 µm) with or without RT co‐treatment. I) The effect of IR‐LND@Lip and RT on T cell killing of tumor cells was evaluated. Data were demonstrated as mean ± SD. Statistical analysis was performed via the two‐tailed Student's *t*‐test. ^*^ *p* < 0.05, ^**^ *p* < 0.01, and ^***^ *p* < 0.001.“(+ RT)” represented the tumor cells treated with RT.

**Figure 3:**
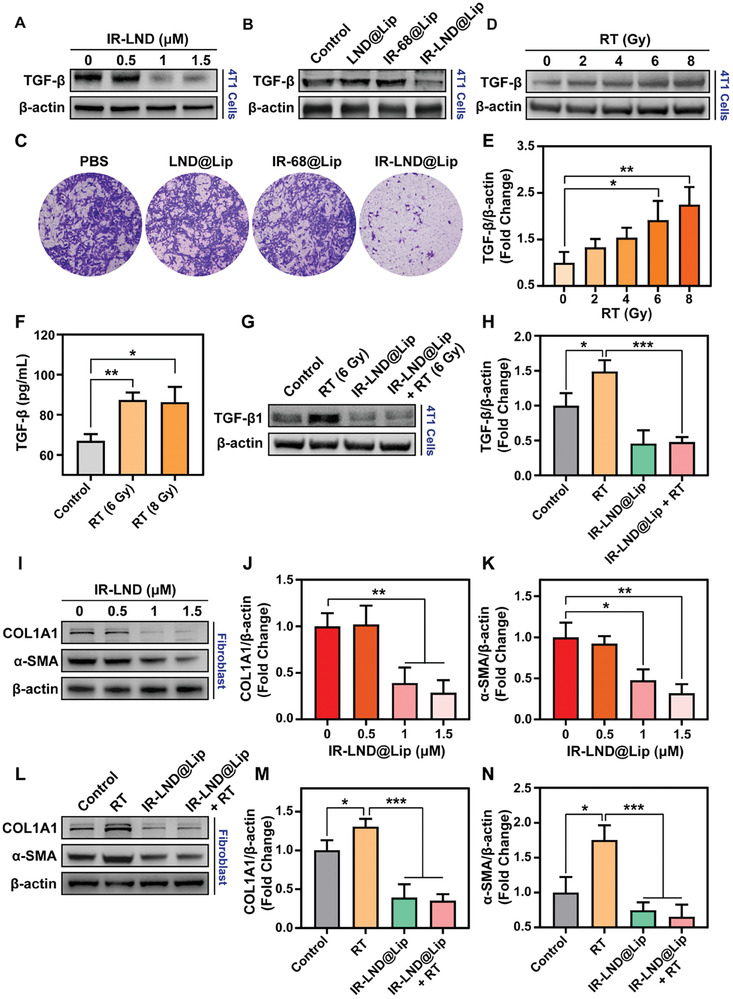
IR‐LND@Lip reversed the radiation‐induced fibrotic tumor microenvironment by downregulating the expression of TGF‐β. A) Detection of the expression of TGF‐β protein in 4T1 cells by western blotting after treatment with indicated doses of IR‐LND (n = 3). B) Detection of the expression of TGF‐β protein in 4T1 cells by western blotting after different treatments (n = 3). C) Evaluation of the migration of 4T1 cells by the in vitro trans‐well migration assay after different treatments. D,E) Detection of the expression of TGF‐β protein in 4T1 cells by western blotting after treatment with different radiation doses and further quantification by Image J (n = 3). F) Determination of the expression of TGF‐β in 4T1 cells by Elisa after treatment with different radiation doses (n = 3). G,H) Detection of the expression of TGF‐β protein in 4T1 cells by western blotting after different treatments with indicated doses of IR‐LND@Lip (0 or 1 µm) with or without RT co‐treatment and further quantification by Image J (n = 3). I–K) Detection of the expression of COL1A1 and α‐SMA protein in myofibroblasts by western blotting after treatment with different doses of IR‐LND and further quantification by Image J (n = 3). L–N) Detection of the expression of COL1A1 and α‐SMA protein in myofibroblasts by western blotting after indicated treatments and further quantification by Image J (n = 3). Data were demonstrated as mean ± SD. Statistical analysis was performed via the two‐tailed Student's *t*‐test. ^*^ *p* < 0.05, ^**^ *p* < 0.01, and ^***^ *p* < 0.001. “(+ RT)” represented the tumor cells treated with RT.

**Figure 4:**
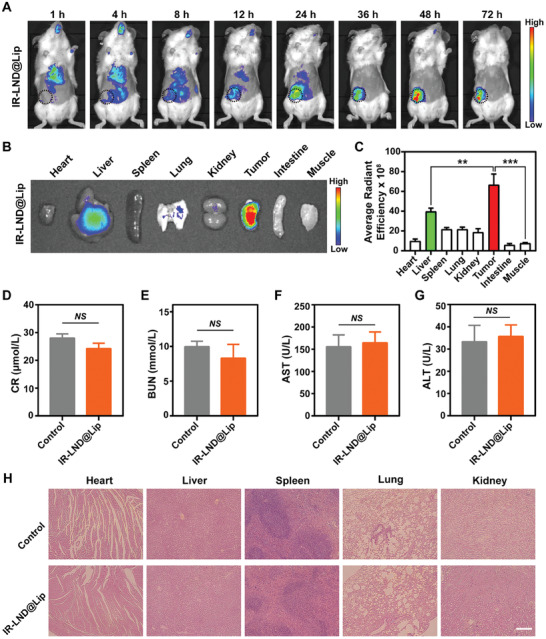
Biodistribution and biosafety evaluation of IR‐LND@Lip nanoparticles. A) The real‐time fluorescence images of 4T1 tumor‐bearing mice were captured at various time points (1, 4, 8, 12, 24, 36, 48, and 72 h) after IR‐LND@Lip administration. The inside of the black dotted circle was the tumor. B,C) Ex vivo fluorescence images of the major organs and tumors at 72 h after treatment with IR‐LND@Lip and further quantified fluorescence intensity (n = 3). D–G) The renal function (CR and BUN) and liver function (AST and ALT) of 4T1 tumor‐bearing mice in two different groups. (n = 3). H) Representative H&E staining images of the major organs collected from the mice treated by Vehicle or IR‐LND@Lip, scale bar = 100 µm. Data were demonstrated as mean ± SD. Statistical analysis was performed via the two‐tailed Student's *t*‐test. ^**^ *p* < 0.01, and ^***^ *p* < 0.001. *NS* indicates no statistically significant difference (*p* > 0.05).

**Figure 5:**
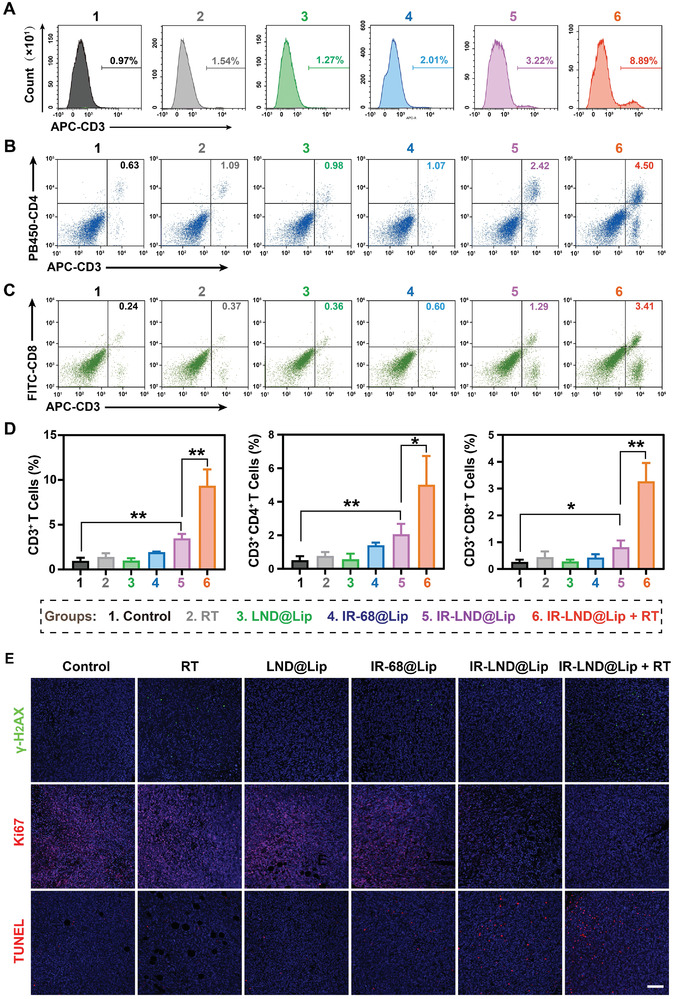
IR‐LND@Lip enhanced the efficacy of radio‐immunotherapy in vivo. A–D) Quantification of CD3^+^, CD4^+^, and CD8^+^ T cells in 4T1 tumors after various treatments using flow cytometry (n = 3). E) Representative fluorescence images of γ‐H_2_AX, TUNEL, and Ki67 in 4T1 tumors after indicated treatments, scale bar = 100 µm. Data were demonstrated as mean ± SD. Statistical analysis was performed via the two‐tailed Student's *t*‐test. ^*^ *p* < 0.05, ^**^ *p* < 0.01, and ^***^ *p* < 0.001. “(+ RT)” represented the tumors treated with RT.

**Figure 6:**
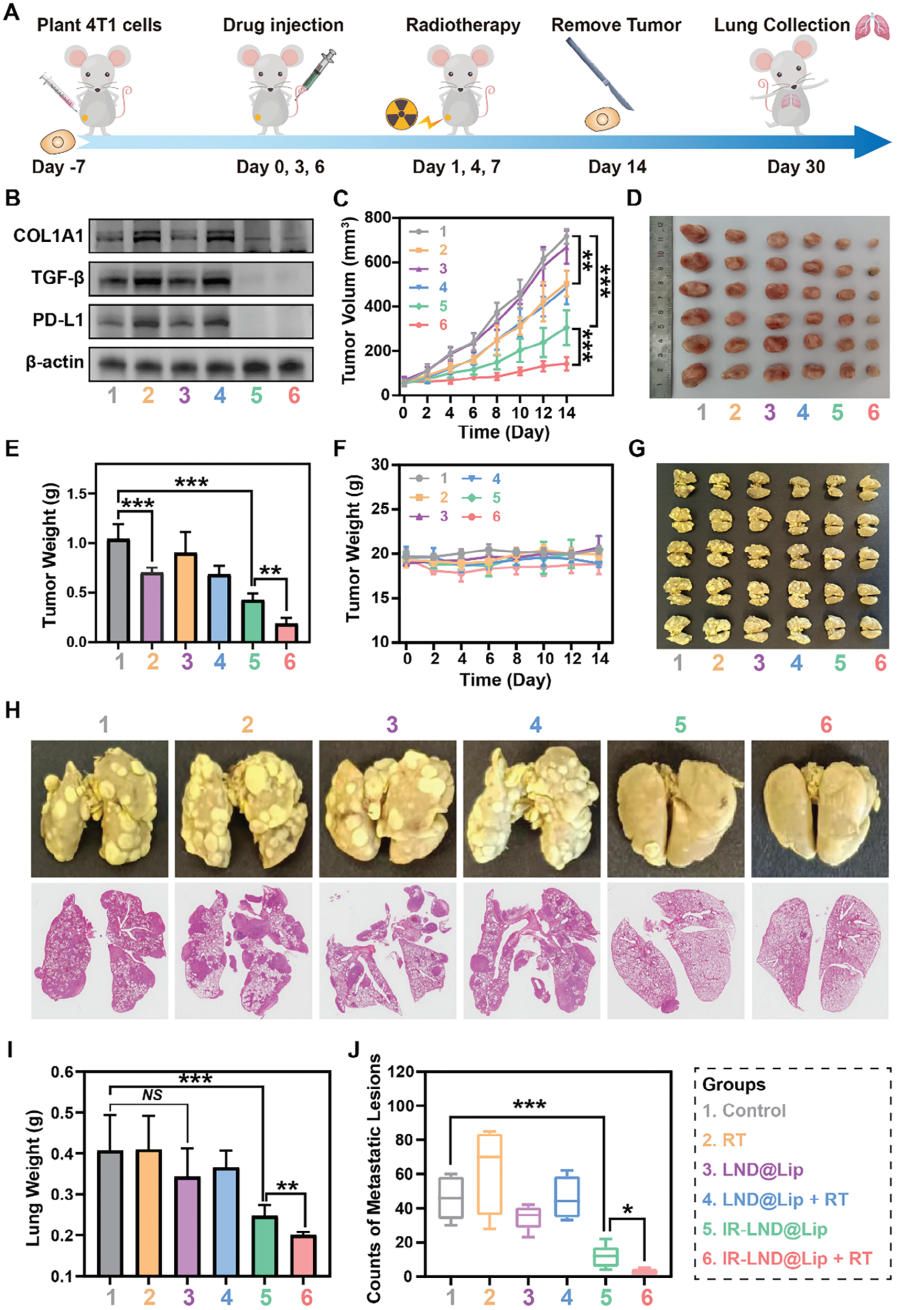
The combination therapy of IR‐LND@Lip and RT markedly suppressed primary tumor growth and tumor metastasis in vivo. A) The schematic diagram of animal experimental design. B) Detection of the expression of PD‐L1, TGF‐β, and COL1A1 proteins in 4T1 tumors by western blotting after different treatments (n = 3). C) Growth curves of the primary 4T1 tumor volume after different treatments within 14 days (n = 6). D) Photographs of primary 4T1 tumors from different groups collected on day 14 (n = 6). E) Tumor weight of collected 4T1 tumors after different treatments on day 14 (n = 6). F) Body weight changes of 4T1 tumor‐bearing mice during the treatment period (n = 6). G) Photographs of 4T1 lung metastasis from different groups collected on day 14 (n = 6). H) Photographs depicting the morphology of lung metastases and histological staining with H&E. I) Quantification of lung weight in 4T1 tumor‐bearing mice after different treatments (n = 6). J) Quantification of lung metastatic nodules in 4T1 tumor‐bearing mice after different treatments (n = 6). Data were demonstrated as mean ± SD. Statistical analysis was performed via the two‐tailed Student's *t*‐test. ^*^ *p* < 0.05, ^**^ *p* < 0.01, and ^***^ *p* < 0.001. *NS* indicates no statistically significant difference (*p* > 0.05). “(+ RT)” represented the tumors treated with RT.

Lonidamine (LND) could be used as a mitochondria complex I and II inhibitor to inhibit the OXPHOS process.^[^ ^13^ ^]^ Thus, LND could work as an OXPHOS depressor to inhibit collagen, TGF‐β, and PD‐L1 expression simultaneously. However, the high dosage needed of LND for effective OXPHOS inhibition, as well as the lack of tumor‐targeting capacity of LND, made it hard to selectively and effectively depress OXPHOS in solid tumors.^[^ ^14^ ^]^ Considering the proven fact that targeting LND to mitochondria via chemical conjugation LND with mitochondria‐targeting triphenylphosphine (TPP^+^) could decrease the dosage of LND in inhibiting tumor growth, IR‐LND was prepared by chemical conjugation the tumor mitochondria‐targeted heptamethine cyanine dye IR‐68 with LND and then was encapsulated with liposomes to form IR‐LND@Lip nanoadjuvants (**Scheme** **1**).^[^ ^13a^ ^]^ IR‐LND@Lip more effectively activated AMPK protein by inhibiting OXPHOS (2 µm IR‐LND vs 300 µm LND), which then effectively, economically, and easily inhibited collagen, TGF‐β, and PD‐L1, as well as reversing tumor hypoxia simultaneously (**Figures** **1**, **2**, and **3**). By doing this, IR‐LND@Lip effectively sensitized RT in vitro by amplifying DNA damage and impairing the DNA damage process by  offering enough oxygen and decreasing PD‐L1 expression (Figure 2). Then, IR‐LND@Lip more effectively amplified CD3^+^, CD4^+^, and CD8^+^ T cell infiltration and activation through PD‐L1, collagen, and TGF‐β co‐inhibition (**Figures** **4** and **5**). All in all, the combination therapy of RT and IR‐LND@Lip finally almost totally inhibited local 4T1 tumor growth and depressed lung metastasis, expanding the clinical application field of mitochondria OXPHOS disruption nanosystem for tumor therapy sensitization including but not only RT (**Figure** **6**).

## 2 Experimental Section

### 2.1 Materials

Unless otherwise specified, all chemical reagents were obtained from Shanghai Aladdin Reagent Co., Fetal bovine serum (FBS), Ltd. RPMI 1640 medium, and trypsin‐EDTA were procured from GIBCO‐BRL (Grand Island, NY, USA). Western blotting reagents were sourced from Beyotime Biotechnology Co., Ltd (Shanghai, China). The origins of all antibodies were specified in their respective sections. TGF‐β1 ELISA kits were bought from NeoBioscience Technology Co., Ltd. Biochemical reagents were used directly without additional purification or modification unless explicitly indicated.

### 2.2 Synthesis of LND@Lip, IR‐68@Lip, and IR‐LND@Lip

The synthesis and validation of IR‐68 and IR‐LND were detailed in our previous studies.^[^ ^15^ ^]^ The synthesis process of liposome nanoadjuvants was as follows: Briefly, 70 mg HSPC, 30 mg cholesterol, 10 mg DSPE‐PEG2000, and 5 mg of IR‐LND were fully dissolved in chloroform. Next, the solution underwent evaporation on a rotary evaporator under negative pressure at 45 °C for 30 min, resulting in the formation of a lipid dry film in a round‐bottom flask. Following this, 4 mL of distilled water was introduced to the dry lipid film, followed by 10 min of sonication in an ultrasonic machine to produce the liposome emulsion. Subsequently, the prepared liposomes were repeatedly squeezed back and forth for 10 cycles using a LiposoFastTM liposome extruder (LiposoFastTM, Avestin, Canada) equipped with a 200 nm polycarbonate membrane (Merck Millipore Ltd., USA), and then these squeezed liposome solutions were placed in an Amicon® Stirred Cells with a 30 kDa molecular weight cutoff membrane. Pressurized rotational ultrafiltration was then performed to remove the free drug (until the ultrafiltrate was colorless), resulting in IR‐LND@Lip nanoparticles. The prepared samples (≈5 mL) were stored in a refrigerator at 4 °C for subsequent experimental use. Additionally, the preparation methods for blank liposomes (Blank Lip), liposomes loaded with IR‐68 (IR‐68@Lip), and liposomes loaded with LND (LND@Lip) were similar to the aforementioned procedure, with ensured drug dosages equivalent to that of IR‐LND@Lip.

### 2.3 Particle Size and Zeta‐Potential Analysis of the Liposomes

The size distribution and zeta potential of Blank Lip, IR‐68@Lip, LND@Lip, and IR‐LND@Lip nanoparticles were measured by DLS (Zetasizer Nano ZS ZEN3600). In brief, equal volumes of the diluted solutions of Blank Lip, IR‐68@Lip, LND@Lip, and IR‐LND@Lip were prepared using distilled water to achieve suitable concentrations. Subsequently, 1 mL of each of these diluted liposomal solutions was introduced into the size analysis chamber, with measurements performed three times to collect data, resulting in the particle size distribution for each category of liposomal nanoparticles. Similarly, 1 mL of each diluted solution was introduced into the zeta potential measurement chamber, and measurements were conducted three times to gather data, yielding the zeta potential distribution for each group of liposomal nanoparticles.

### 2.4 Morphology Characterization of the Liposomes

The morphology of IR‐LND@Lip nanoparticles was investigated using Transmission Electron Microscopy (TEM). The procedure was as follows: First, an appropriate dilution of the IR‐LND@Lip solution was prepared. Next, a drop of the diluted liposome solution was carefully placed onto a copper grid using a 1 mL syringe. After allowing the sample to naturally air‐dry, a 1% solution of phosphotungstic acid negative stain was gently applied for 1 min. Any excess moisture was carefully blotted away using filter paper, followed by three rinses with PBS. Subsequently, the copper grid was positioned in a 40 °C oven for 48 h to facilitate thorough drying. Ultimately, the TEM technique was employed to observe and analyze the nanoparticles’ morphology.

### 2.5 Detection of the content of IR‐LND

First, 2 mg of IR‐LND was precisely weighed and fully dissolved in 1 mL of ethanol, and then diluted by concentration gradient to obtain IR‐LND solutions with concentrations of 0.5, 1, 2, and 4 µg mL^−1^, respectively. Following this, the ultraviolet absorption spectrum was measured using a UV–NIR spectrophotometer, with absorbance recorded at 780 nm. A standard curve was then constructed, with the concentration of IR‐LND on the X‐axis and the absorbance at 780 nm on the Y‐axis. Finally, the IR‐LND@Lip solution was demulsified with ethanol, and the drug loading (DL, %) and encapsulation efficiency (EE, %) of IR‐LND in liposomes were calculated based on the standard curve. Moreover, the process of constructing standard curves for IR‐68 and LND, coupled with drug content computations within liposomes, follows a parallel protocol. The DL and EE of IR‐68, LND, and IR‐LND were assessed employing the formulas:

(1) $$\mathrm{DL}\left(\%\right)=\left(W_{\mathrm{drug}}/W_{\mathrm{lipsomes}}\right)\times 100\%$$

(2) $$\mathrm{EE}\left(\%\right)=\left(W_{\mathrm{drug}}/W_{\mathrm{Total}}\right)\times 100\%$$

wherein *W*_drug_ signifies the measured IR‐LND drug content within the liposome assembly; *W*_lipsomes_ embodies the collective mass of all drugs encapsulated within the liposomes; *W*_Total_ represents the original mass of the introduced IR‐LND.

Notably, IR‐LND@Lip exhibited an encapsulation efficiency of 83.89% alongside a drug loading of 3.65%. For IR‐68@Lip, the encapsulation efficiency was 87.14%, concomitant with a drug loading of 3.79%. As regard LND@Lip, the encapsulation efficiency stood at 48.05%, alongside a drug loading of 2.09%.

### 2.6 Stability Testing of IR‐LND@Lip

IR‐LND@Lip (1 mL) was diluted 200 times with PBS at room temperature. On days 0, 2, 4, 6, and 8, 1 mL of the diluted solution was subjected to three repetitions of particle size analysis using a particle size analyzer, with the average particle size being recorded.

### 2.7 Cell Culture

The murine bladder cancer cell line MB49 and the triple‐negative breast cancer cell line 4T1 were obtained from the American Type Culture Collection. These cell lines were cultured in RPMI 1640 medium supplemented with 10% FBS and 1% penicillin/streptomycin. The tumor cells were incubated in a cell culture incubator maintained at 37 °C with 5% CO_2_.

### 2.8 Subcellular Localization of IR‐LND@Alb

Concisely, 1.5 × 10^5^ 4T1 cells were plated in glass‐bottom cell culture dishes. Following a 12‐h incubation period, tumor cells were subjected to a 4‐h exposure to either 1 µm IR‐68@Lip or IR‐LND@Lip (Calculated by IR‐68 or IR‐LND concentration). Subsequently, 4T1 cells were treated with MitoTracker Green (100 nm) for 30 min. Following three washes with PBS, colocalization of the red fluorescence emitted by IR‐LND@Lip and the green fluorescence originating from labeled mitochondria by MitoTracker Green was observed using confocal laser scanning microscopy (CLSM).

### 2.9 Measurement of Intracellular Oxygen Consumption Rate

The Seahorse XF96 (Agilent Technologies) was employed to measure the intracellular oxygen consumption rate (OCR). Initially, 4T1 cells were cultured in Seahorse XF cell culture microplates (96 wells) to an appropriate cell density. Subsequently, the cells were treated with LND@Lip (2 µm or 200 µm), IR‐68@Lip (2 µm), or IR‐LND@Lip (2 µm) (Calculated by LND, IR‐68, or IR‐LND concentration) for 8 h respectively. Following this treatment, the culture medium was replaced with preheated assay medium (containing 2 mmol L^−1^ glutamine, 1 mmol L^−1^ sodium pyruvate, and 10 mmol L^−1^ glucose in complete culture medium), and the cultivation of the cells was continued for an additional 1 h. Working solutions containing compounds (oligomycin, FCCP, rotenone/antimycin A) were prepared and added to the drug addition ports in the probe plate. Calibration plates and drug‐treated probe plates were placed in the instrument tray for baseline calibration. The experiment was run by replacing the calibration plate with the cell culture microplate, resulting in the Seahorse XF mitochondrial stress test report.

### 2.10 Western Blot Analysis

First, 1 × 10^6^ MB49 or 4T1 cells were seeded and incubated in the 6‐cm cell culture dishes for 24 h and treated with IR‐LND@Lip, LND@Lip, IR‐68@Lip, or fresh medium for an additional 24 h respectively. Then, the cells in the RT group were cultured for 24 h after exposure to 6 Gy of radiation, and the cells in the non‐RT group were also cultured at the same time. Subsequently, the cells were washed with PBS, and cell lysate containing phosphatase inhibitors and protease inhibitors was added to lyse the cells for 20 min. Subsequently, the cells were washed three times with PBS, cell lysis solution (RIPA) containing phosphatase inhibitors and protease inhibitors was added to lyse the cells for 20 min, and then the cell lysate was collected and centrifuged in a 1.5 mL centrifuge tube at 4 °C and 12 000 rpm for 20 min. Next, the protein supernatant was collected, and the total protein concentration of each sample was determined using the BCA protein assay kit. The samples were diluted with PBS to maintain the same total protein concentration. Following this, the protein loading buffer was added, and the protein was denatured by heating at 100 °C for 10 min.^[^ ^16^ ^]^

Routinely, equal amounts of protein were separated by 10% SDS‐polyacrylamide gels and transferred to the PVDF membrane. The membranes were then blocked with 5% fat‐free milk and incubated overnight at 4 °C with primary antibodies, such as β‐actin antibody (1: 5000, Affinity), rabbit anti‐mouse CD274 antibody (1: 1000, Affinity), rabbit anti‐mouse HIF‐1α antibody (1: 1000, Affinity), rabbit anti‐mouse TGF‐β (1: 1000, CST), rabbit anti‐mouse COL1A1 antibody (1: 1000, CST), rabbit anti‐mouse p‐AMPK (1:1000, CST), rabbit anti‐mouse AMPK (1:1000, CST) and rabbit anti‐mouse alpha‐smooth muscle actin antibody (1: 1000, CST). Following three washes with TBST solution, the membranes were incubated with secondary antibodies for 2 h at room temperature. Ultimately, protein band images were visualized using a Fluorescent and chemiluminescence Gel Imaging System (Peiqing Science and Technology, Shanghai, China) and quantified using ImageJ.^[^ ^17^ ^]^

### 2.11 Effects of IR‐LND@Lip on the Expression of AMPK, pAMPK, TGF‐β, and PD‐L1 In Vitro

In short, 1 × 10^6^ 4T1 cells were seeded in 6 cm cell culture dishes for 12 h. Afterward, the cells were subjected to a 24‐h incubation period with 3 mL of fresh culture medium containing varying concentrations of IR‐LND@Lip (0, 0.5, 1, or 1.5 µm). After this, proteins were extracted from different cell groups, and the expression levels of AMPK, pAMPK, and β‐actin proteins were assessed via western blot analysis. Quantification of the results was then carried out using ImageJ.

Furthermore, 1 × 10^6^ 4T1 cells were plated in 6 cm cell culture dishes. Following a 12‐h incubation period, the cells received additional treatment with 3 mL of fresh culture medium containing either 10 µm Compound C, 1.5 µm IR‐LND@Lip, or a combination of 10 µm Compound C + 1.5 µm IR‐LND@Lip for an additional 24 h. Cells treated with PBS were used as the control group. Subsequently, the expression levels of AMPK, pAMPK, PD‐L1, and β‐actin proteins in these cells were assessed via western blot, followed by further quantification using ImageJ.

### 2.12 Real‐Time Quantitative PCR (RT‐qPCR)

Briefly, 1 × 10^6^ MB49 or 4T1 cells were seeded and incubated in the 6‐cm cell culture dishes for 24 h. Next, 1 µm IR‐LND@Lip (Calculated by IR‐LND concentration) was added to one group, while the other group was not treated. After 24 h, the cells were replaced with a new culture medium, irradiated with 6 Gy X‐rays, and cultured for another 24 h. Total RNA in the cells was extracted according to the instructions of the TRIzol reagent (Invitrogen, Carlsbad, CA, USA), and reverse transcription was performed using a reverse transcription kit (#RR047A, Takara, Japan). Finally, the mRNA expression level was evaluated using a real‐time quantitative PCR instrument following the instructions of the PCR kit (#RR820A, Takara, Japan). The primer sequences for RT‐PCR are shown in **Table** **1**.

**Table 1:** The forward primer and reverse primer RNA sequence used for the detected genes.

Gene (Mouse)	Forward primer (5′ – 3′)	Reverse primer (5′ – 3′)
β‐actin	GGCTGTATTCCCCTCCATCG	CCAGTTGGTAACAATGCCATGT
NBS1	GCAGAAGACGACGAAGAGGAACAG	GCCAATCCAATCTCCGCTTCAGG
MRE11	TCGGGCACAACATCTAGCAAACG	GAAGCAAAACCGGACTAATGTCT
RAD50	CGATGCAGTGCTGGACAGAAGG	AGACTGACCTTTTCACCATGC

### 2.13 γ‐H2AX Immunofluorescence Analysis

Generally, 1 × 10^6^ 4T1 or MB49 cells were seeded in a confocal dish and cultured overnight. Subsequently, the cells were incubated with fresh media containing PBS or IR‐LND@Lip (1 µm) (Calculated by IR‐LND concentration) for 24 h respectively. The cells in the RT group were put back into the incubator after being treated with 6 Gy X‐ray radiation, and all the cells were cultured for an additional 24 h. Following this, the cells were fixed with 4% paraformaldehyde for 15 min, punched with 0.3% Triton X‐100 for 30 min, blocked with 3% BSA for 1 h at room temperature and then incubated overnight with γ‐H_2_AX antibody (1: 200, Affinity) at 4 °C. After washing with PBS three times, they were then incubated with fluorescently labeled secondary antibody (Alexa Fluor™ 488, Thermo Fisher) for 1 h at room temperature. Ultimately, the nuclei were stained with DAPI for 10 min and fluorescence images were performed by using CLSM.

### 2.14 CCK‐8 Assay

In brief, 4T1 cells were seeded in 96‐well plates at a density of 5×10^3^ cells per well overnight and subsequently incubated with various concentrations of IR‐LND@Lip (0, 1.5, 2, or 3 µm) (calculated based on IR‐LND concentration) for 24 h. Next, the cells in the RT group were put back into the incubator after being treated with 6 Gy X‐ray radiation, and all the cells were cultured for another 24 h. Ultimately, cell viability was assessed using the CCK‐8 assay kit (Beyotime Biotechnology Co., Ltd, Shanghai, China) following the manufacturer's instructions.

### 2.15 Clonogenic Assay

Culturing 5×10^4^ 4T1 cells in a 24‐well plate overnight. Then, the cells were pre‐treated with fresh medium containing PBS or IR‐LND@Lip (1 µm) (Calculated by IR‐LND concentration) for 24 h respectively. Subsequently, the fresh culture medium was replaced, and the cells in the radiation group underwent 6 Gy X‐ray radiation before being returned to the incubator. After 24 h, the cells were digested and counted with trypsin. 1000 cells from each group were transferred to a 6‐well plate for an additional 7‐day incubation. Finally, the cells were stained with crystal violet for 10 min, washed with PBS three times, and images were taken to record the cloning status in each well.

### 2.16 T cell‐Mediated Tumor Cell Killing Assay

From a healthy individual, 10 mL of venous blood was drawn. Lymphocytes were separated using a lymphocyte isolation tube and centrifuged. The Peripheral Blood Mononuclear Cells (PBMC) layer was aspirated in a biosafety cabinet and washed twice with RPMI 1640 medium. Human primary T cells were isolated using the EasyStep™ Human T Cell Isolation Kit (Stemcell) and a magnet (Stemcell). Next, the T cells were placed in ImmunoCult™‐XF T Cell Expansion Medium (Stemcell) and activated with Phorbol‐12‐myristate‐13‐acetate (PMA, 50 ng mL^−1^, MedChemExpress) and ionomycin (500 ng mL^−1^, MedChemExpress) for 12 h. Subsequently, the T cell expansion medium containing Recombinant Human IL‐2 (10 ng mL^−1^) was used to expand the T cells to the desired quantity. Human bladder cancer cells (5637 cells) were pre‐treated with IR‐LND@Alb (1 µm) (Calculated by IR‐LND concentration) for 24 h. Then, an equal number of these cells were then cultured in a 12‐well plate for 4 h. Following this, the activated T cells and tumor cells were co‐cultured at a ratio of 10:1 (T cells: tumor cells) in RPMI 1640 medium supplemented with 10% FBS, 1% penicillin/streptomycin, 100 ng mL^−1^ Anti‐mouse CD3e, and 10 ng mL^−1^ IL‐2 for 48 h. After that, suspended T cells and tumor cell debris were washed away with PBS and the adherent tumor cells were stained with crystal violet and photographed. Finally, the crystal violet within the cells was dissolved in 33% acetic acid, and the absorbance was measured at 570 nm.

### 2.17 Transwell Assay

4T1 cells (5×10^5^) were cultured in a 6‐well plate and incubated overnight. Subsequently, the cells were separately treated with fresh media containing PBS, LND@Lip (1 µm), IR‐68@Lip (1 µm) and IR‐LND@Lip (1 µm) for 24 h (Calculated by LND, IR‐68, or IR‐LND concentration). After digesting the cells with trypsin and counting, 5×10^4^ cells from each group were placed in Transwell chambers, ensuring that the chambers contained 0.2 mL of serum‐free culture medium, while 0.6 mL of RPMI 1640 medium containing 10% FBS was added to the lower chamber. The setup was placed in an incubator and incubated for an additional 24 h. The culture medium from both the upper and lower chambers was aspirated using a pipette tip, then fixed with 4% paraformaldehyde for 15 min, and stained with crystal violet staining solution for 10 min. At last, the staining solution was discarded, and the cells were washed twice with PBS, air‐dried, and photographed under a microscope.

### 2.18 Determination of Intracellular TGF‐β Content

Briefly, 1×10^6^ 4T1 cells were seeded in a 6‐well plate and incubated overnight. After irradiation with 6 Gy or 8 Gy X‐ray, the cells were further cultured for 24 h. Subsequently, the cells were digested with trypsin, washed twice with PBS, and then diluted in PBS to achieve a cell concentration of 1×10^6^ cells mL^−1^. Following this, the cell suspensions were subjected to five cycles of freezing and thawing at −20 °C. Afterward, the suspensions were centrifuged for 20 min at 2000–3000 rpm, and the supernatant was collected. At last, the TGF‐β1 concentration was determined following the instructions of a mouse TGF‐β1 ELISA kit (NeoBioscience Technology Co., Ltd).

### 2.19 Inducing Fibroblasts into Cancer‐Associated Fibroblasts

In short, 4T1 cells in the logarithmic growth phase were subjected to 6 Gy X‐ray radiation and then cultured for an additional 24 h. Next, the culture medium was collected, and centrifuged for 20 min at 2000–3000 rpm, and the supernatant was preserved. After passing through a 220 µm filter, it was stored in a 4 °C refrigerator. In the first group, embryonic fibroblasts (NIH/3T3) were cultured in a complete culture medium containing 20% FBS. In the second group, the NIH/3T3 cells were cultivated in a conditional medium (CM). CM is a blend of a complete medium comprising 20% FBS and culture medium derived from irradiated 4T1 cells, mixed at a 1:1 ratio. Under this specific culture condition, the NIH/3T3 cells underwent differentiation, transforming into myofibroblasts. In the third group, the cells received conditional culture medium and were exposed to 6 Gy X‐ray radiation. All the cells were continuously cultured in the incubator for 5 days. Subsequently, cellular proteins were extracted, and western blot analysis was employed to detect the expression of α‐SMA protein, and the secreted COL1A1 protein.

### 2.20 In Vivo Imaging

The ethical approval for this experiment was obtained from Wenzhou Institute, University of Chinese Academy of Sciences ([Issue NO.]: WIUCAS22093001). Balb/c mice weighing 18–20 g were purchased from the Zhejiang Experimental Animal Center. A suspension of 4T1 cells (1×10^6^ cells, 100 µL) was prepared and injected subcutaneously into the upper left hind limb of the Balb/c mice. The mice were then raised in a specific‐pathogen‐free environment for approximately 10 days. When the 4T1 tumor volumes reached 200 mm^3^, the tumor‐bearing mice were intravenously injected with 100 µL of IR‐LND@Lip (0.8 mg mL^−1^) (Calculated by IR‐LND concentration). At different time points after administration (1, 6, 12, 24, and 48 h), the mice were anesthetized with isoflurane and placed in the in vivo fluorescent imaging system (IVIS Lumina XRMS Series III) to observe the fluorescence distribution of IR‐LND@Lip within their bodies. After 72 h, the mice were euthanized, and both the tumors and normal tissues (heart, spleen, lung, liver, kidney, muscle, and small intestine) were extracted and subjected to ex vivo fluorescence imaging.

### 2.21 Biosafety Assessment of IR‐LND@Lip

Healthy Balb/c mice were divided into two groups (n = 3): Vehicle, or IR‐LND@Lip. The mice were intravenously injected with the drugs (IR‐LND@Lip at 0.8 mg mL^−1^) or an equivalent volume of saline solution (Vehicle group) (Calculated by IR‐LND concentration). The first dosing was on day 0, followed by dosing on days 3 and 6. On day 14, all the mice were sacrificed to obtain the blood and major organs (heart, liver, spleen, lung, kidney). The concentrations of creatinine (CR), blood urea nitrogen (BUN), alanine aminotransferase (ALT), and aspartate aminotransferase (AST) in the collected serum samples were measured following the instructions in the assay kits (Nanjing Jiancheng Bioengineering Institute, China) to assess the hepatorenal toxicity of these treatments. Additionally, the collected main organs were fixed in 4% neutral formalin, embedded in paraffin, and stained with H&E for histological analysis.

### 2.22 Western Blot In Vivo

Briefly, 4T1 tumor‐bearing mice (≈100 mm^3^) were randomly divided into six groups (n = 3): Vehicle, Vehicle + RT, LND@Lip, LND@Lip + RT, IR‐LND@Lip, and IR‐LND@Lip + RT. The mice were intravenously injected with the drugs (LND@Lip or IR‐LND@Lip at 0.8 mg mL^−1^) or an equivalent volume of saline solution (Vehicle group) (Calculated by LND, or IR‐LND concentration). The drugs were administered on days 0 and 3, and the tumor sites of the mice in the RT groups were irradiated with 6 Gy X‐rays on days 1 and 4. On day 6, all the mice were euthanized and the tumor tissues were extracted to measure the expression of TGF‐β, PD‐L1, and COL1A1 by Western blot. The method of immunoblotting was the same as before, and the primary antibodies used were as follows: rabbit anti‐mouse COL1A1 antibody (1:1000, CST), rabbit anti‐mouse CD274 antibody (1:1000, Affinity), rabbit anti‐mouse TGF‐β (1:1000, CST), and rabbit against mouse β‐actin antibody (1:5000, Affinity).

### 2.23 In Vivo Antitumor Efficacy of IR‐LND@Lip and Radiation Therapy

To validate the effectiveness of IR‐LND@Lip in alleviating T cell exhaustion, thereby enhancing the efficacy of radio‐immunotherapy in vivo, 4T1 tumor‐bearing (∼200 mm^3^) mice were randomly divided into six groups (n = 3): Vehicle, Vehicle + RT, LND@Lip, IR‐68@Lip, IR‐LND@Lip, and IR‐LND@Lip + RT. The mice were intravenously injected with the drug (LND@Lip, IR‐68@Lip, or IR‐LND@Lip at 0.8 mg mL^−1^) or an equivalent volume of saline solution. 24 h later, the mice in the RT groups were subjected to 6 Gy X‐ray irradiation at the tumor site. After another 48 h, all the mice were euthanized and the tumor tissues were collected for flow cytometry analysis, immunofluorescence staining, and H&E staining.

Furthermore, the 4T1 subcutaneously transplanted tumor model and metastatic tumor model were employed to assess the antitumor efficacy of IR‐LND@Lip mediated RT immunotherapy. In short, when the tumor volume reached 100 mm^3^, the 4T1 tumor‐bearing mice were randomly divided into six groups (n = 6): Vehicle, Vehicle + RT, LND@Lip, LND@Lip + RT, IR‐LND@Lip, and IR‐LND@Lip + RT. The mice were intravenously injected with the drug (LND@Lip or IR‐LND@Lip at 0.8 mg mL^−1^) or an equivalent volume of saline solution (Calculated by LND or IR‐LND concentration). The first administration time was specified as day 0, and administration was carried out on days 0, 3, and 6. 24 h after each administration, the mice in the RT groups were subjected to 6 Gy X‐ray irradiation at the tumor site. Tumor dimensions and body weight were measured every two days using calipers until day 14. Subsequently, all the mice were anesthetized, all 4T1 tumor tissues were excised and the wounds were sutured. The excised tumor tissues were collected, weighed, and photographed. On day 30, all the mice were euthanized, and intact lung tissues were immersed in a 4% paraformaldehyde solution containing 0.3% saturated picric acid. Next, the lung tissues were arranged according to their respective groups, and the number of lung nodules was recorded. Finally, the lung tissues were prepared into paraffin sections for H&E staining.

### 2.24 Flow Cytometric Analysis

After different treatments, the 4T1 tumor tissues were harvested, ensuring they were of the same size. These tumor tissues were placed into a single‐cell suspension preparation device to obtain a cell suspension, which was then transferred to 2 mL centrifuge tubes. The tubes were centrifuged at 4 °C for 5 min at 2000 rpm, and the supernatant was discarded. The cells were washed twice with PBS (centrifugation at 2000 rpm, 4 °C, 5 min), and the supernatant was discarded each time. The collected cells were resuspended in PBS and counted. An equivalent number of tumor cells from each group were taken and mixed with an appropriate amount of flow cytometry dye, followed by incubation at 4 °C for 30 min. The cells were then centrifuged to remove excess dye. At last, the cells were resuspended in PBS for flow cytometry analysis, with a controlled flow rate of approximately 500 cells per second.

### 2.25 Immunofluorescence Staining of Tumor Tissues

After different treatments, the 4T1 tumor tissues were embedded in OCT compound, frozen at −20 °C, and then sectioned into 6 mm‐thick slices and fixed in cold acetone. Following this, the slices were fixed in 4% paraformaldehyde for 15 min, treated with Triton X‐100 for 30 min, blocked with 3% BSA for 1 h at room temperature, and then incubated with fluorescent antibodies for 1 h. The antibodies used were the rabbit anti‐mouse γ‐H_2_AX antibody, rabbit anti‐mouse Ki67 antibody, and TUNEL Cell Apoptosis Detection Kit (red fluorescence). Next, the slices were gently washed with PBS and stained with DAPI. Finally, immunofluorescence imaging of the tumor tissue sections was observed using CLSM.

### 2.26 Statistical Analysis

Statistical analysis and plotting were performed using GraphPad Prism 9.0. All results were shown as means ± standard deviation of three independent experiments. Two‐group comparisons were conducted using a two‐tailed Student's *t*‐test, and multiple‐group comparisons were assessed using a one‐way ANOVA test. ^*^ *p* < 0.05 was considered statistically significant; ^**^ *p* < 0.01 and ^***^ *p* < 0.001 were considered highly significant; *NS* indicates no statistically significant difference (*p* > 0.05).

## 3 Results

### 3.1 Preparation and Characterization of the IR‐LND@Lip

As previously reported, LND could be used as an inefficient mitochondria complex I and II inhibitor to inhibit the OXPHOS process, which may possess the capacity to depress multi‐immune regulation proteins like collagen, PD‐L1, and TGF‐β, simultaneously.^[^ ^13a^ ^]^ But, at present, no research has revealed whether LND possesses such capacity. To prove this, in this study, we newly revealed that LND could inhibit collagen, and TGF‐β together at a high dosage in vitro in 4T1 tumor cells, as well as the just previously proven function of LND in PD‐L1 depression by us before (Figure S1, Supporting Information).^[^ ^13a^ ^]^ Even so, the poor efficiency and no‐tumor targeting capacity of LND still limited its possible usage as a multi‐functional immune regulator.^[^ ^11^, ^13^ ^]^ Considering the proven fact that targeting LND to mitochondria via chemical conjugation LND with mitochondria‐targeting triphenylphosphine (TPP^+^) could decrease the dosage of LND in inhibiting tumor growth, IR‐LND was prepared by chemical conjugation the tumor mitochondria‐targeted heptamethine cyanine dye IR‐68 with LND and then was encapsulated with liposomes to form IR‐LND@Lip nanoadjuvants (Scheme 1A; Figure S2, Supporting Information).^[^ ^13a^ ^]^ As shown in Figure 1A, IR‐LND@Lip nanoadjuvants were synthesized via a thin‐film hydration method, and IR‐LND was encapsulated in the hydrophobic layer of the liposome.^[^ ^18^ ^]^ TEM analysis showed that IR‐LND@Lip exhibited a spherical structure and had an average size of approximately 200 nm, correspondence with the results obtained from dynamic light scattering measurements (Figure 1B,C). Blank Lip, IR‐68@Lip, and LND@Lip possessed nearly the same size of about 200 nm (Figure S3, Supporting Information). Meanwhile, the zeta potentials were −23.8 ± 3.5, −10.9 ± 2.1, −21.5 ± 2.9, and −12.6 ± 1.7 mV for Blank Lip, IR‐68@Lip, LND@Lip, and IR‐LND@Lip, respectively (Figure 1D). Moreover, when IR‐LND@Lip was stored at room temperature for 8 days, its particle size didn't exhibit any significant changes, demonstrating its excellent storage stability (Figure 1E). Additionally, the fluorescence of IR‐68 and IR‐LND was significantly enhanced when they were loaded into liposomes (Figure 1F; Figure S4A, Supporting Information), and typical absorption peaks of IR‐68, LND, and IR‐LND were observed in IR‐68@Lip, LND@Lip, and IR‐LND@Lip respectively (Figure 1G; Figures S4B and S5, Supporting Information). According to the results of UV absorption, we calculated the loading and encapsulation efficiency of IR‐LND, which were 3.65% and 83.9% in IR‐LND@Lip respectively (Figure S6, Supporting Information). All these results indicated that these liposomes were well prepared for the following research.

### 3.2 IR‐LND@Lip Enhanced Radio‐Immunotherapy by Facilitating Oxygen Reversion and Inhibiting PD‐L1 Protein

Considering that LND is capable of reducing ATP production in tumor cells by inhibiting mitochondrial oxidative phosphorylation, consequently leading to the activation of the AMPK protein pathway, we initially investigated the impact of IR‐LND@Lip on mitochondrial function in tumor cells (Figure 2). First, owing to the tumor mitochondria‐targeting ability of the IR‐68, we observed that IR‐68@Lip and IR‐LND@Lip can selectively accumulate within the mitochondria of tumor cells (Figure 2A). Moreover, even at extremely low doses, IR‐LND@Lip was able to significantly inhibit the OCR of tumor cells (Figure 2B) and efficiently downregulate the expression of PD‐L1 and HIF‐1α protein in tumor cells (Figure 2C; Figures S7 and S8, Supporting Information). Moreover, in alignment with our previous study, we observed that IR‐LND@Lip exhibited the capacity to activate AMPK in a dose‐dependent manner (Figure S9, Supporting Information).^[^ ^15^ ^]^ Treatment with Compound C (Com C), an AMPK inhibitor, reversed the AMPK phosphorylation induced by IR‐LND@Lip, thereby alleviating the IR‐LND@Lip‐induced reduction of PD‐L1 expression in 4T1 cells (Figure S10, Supporting Information). This suggested that IR‐LND@Lip could reduce PD‐L1 expression by activating the AMPK pathway. Following this, results revealed that RT could radiation dose‐dependently increase the expression of PD‐L1 protein in tumor cells (Figure S11, Supporting Information). Conversely, IR‐LND@Lip effectively countered this effect mediated by RT (Figure 2D; Figures S12 and S13, Supporting Information) and reduced the expression of the MRE11‐RAD50‐NBS1 complex to inhibit the DNA damage repair process in tumor cells (Figure 2E, Table 1). Additionally, when combined with IR‐LND@Lip, RT led to a notably increased expression of γ‐H_2_AX, a typical marker for DNA damage (Figure 2F). CCK‐8 assays further affirmed that IR‐LND@Lip enhances the cytotoxic effects of RT in a dose‐dependent manner (Figure 2G). Meanwhile, clonogenic assays once again confirmed that the combination of RT with IR‐LND@Lip achieved the most effective growth inhibition of tumor cells (Figure 2H; Figure S14, Supporting Information). Furthermore, we assessed the effectiveness of IR‐LND@Lip in enhancing T cell‐mediated tumor cell elimination. The findings demonstrated that IR‐LND@Lip boosted T cell activation, empowering them to more efficiently eradicate tumor cells. Moreover, when combined with RT, IR‐LND@Lip maximized T cell‐mediated tumor cell killing, possibly owing to its capability to disrupt the PD‐1/PD‐L1 recognition by inhibiting PD‐L1 expression in tumor cells, thus reinvigorating T cell‐mediated immunotherapy (Figure 2I; Figure S15, Supporting Information). In summary, all these findings indicated that IR‐LND@Lip can enhance the effectiveness of radio‐immunotherapy by reversing the tumor's hypoxic microenvironment and reducing the expression of PD‐L1 protein in tumor cells.

### 3.3 IR‐LND@Lip Efficiently Counteracted the Radiation‐Induced Fibrotic Tumor Microenvironment by Downregulating the Expression of TGF‐β

Earlier studies have shown that IR‐LND, equipped with the ability to accumulate in mitochondria, could effectively trigger the AMPK protein pathway.^[^ ^13a^ ^]^ Activating the AMPK pathway has been confirmed to lead to the downregulation of TGF‐β, consequently mediating fibrotic alterations in tumor tissues.^[^ ^11^ ^]^ Consequently, we postulated that IR‐LND and its liposome nanosystem can proficiently diminish the expression of TGF‐β in tumor cells. As expected, our findings revealed that even at exceedingly low doses (1 µm), IR‐LND effectively lowered the expression of TGF‐β in tumor cells (Figure 3A; Figures S16 and S17, Supporting Information). Moreover, when compared to LND@Lip, IR‐LND@Lip exhibited a significantly higher efficacy in reducing the expression of TGF‐β (Figure 3B; Figures S18 and S19, Supporting Information).

In addition, treatment with Com C reversed the AMPK phosphorylation induced by IR‐LND@Lip, consequently relieving the decrease in TGF‐β expression caused by IR‐LND@Lip in 4T1 cells (Figure S10, Supporting Information). This indicated that IR‐LND@Lip could reduce TGF‐β expression through AMPK signaling activation. Further transwell experiments also suggested that IR‐LND@Lip can notably inhibit the invasive capability of tumor cells (Figure 3C). Additionally, in line with prior research findings, our western blotting results revealed that RT triggered a dose‐dependent elevation in TGF‐β expression (Figure 3D,E; Figure S20, Supporting Information). This observation was substantiated by Elisa's results obtained from 4T1 cells (Figure 3F). Remarkably, the co‐treatment of 4T1 or MB49 cells with IR‐LND@Lip and RT demonstrated a significant reversal of the RT‐induced upregulation of TGF‐β by IR‐LND@Lip (Figure 3G,H; Figure S21, Supporting Information). Moreover, we verified that RT triggered an increase in the expression of COL1A1 and α‐SMA in cancer‐associated fibroblasts (CAFs) (Figure S22, Supporting Information). Conversely, IR‐LND@Lip induced a dose‐dependent reduction in COL1A1 and α‐SMA expression in CAFs (Figure 3I–K). Importantly, when fibroblasts were co‐treated with IR‐LND@Lip and RT, IR‐LND@Lip could effectively counteract the RT‐induced upregulation of COL1A1 and α‐SMA expression (Figure 3L–N). In summary, IR‐LND@Lip can effectively alleviate the fibrotic tumor microenvironment induced by RT by downregulating the expression of TGF‐β.

### 3.4 Biodistribution and Biosafety Evaluation of IR‐LND@Lip Nanoparticles

Encouraged by the outstanding tumor mitochondrial‐targeting capability demonstrated by IR‐LND@Lip in vitro, we further assessed the tumor accumulation capacity of IR‐LND@Lip in 4T1 tumor‐bearing mice through a real‐time NIR fluorescence imaging assay. After IR‐LND@Lip administration, the distribution of IR‐LND in tumors was enhanced as time went on, reaching its maximum at 48 h (Figure 4A). Subsequently, all mice were sacrificed, and normal tissues and tumors were collected for ex vivo fluorescence imaging (Figure 4B). It was worth noting that the fluorescence intensity of IR‐LND@Lip in tumors was significantly higher than that in normal organs, even reaching nearly 10 times that of the surrounding muscle tissues (Figure 4C). These results indicated that the IR‐LND@Lip nanosystem possessed outstanding capability for selective accumulation in tumors. Following this, we performed a more comprehensive evaluation of the biocompatibility of IR‐LND@Lip nanoparticles. The findings showed that the liver and kidney function indexes in mice treated with IR‐LND@Lip didn't exhibit significant differences when compared to the control group (Figure 4D–G). Furthermore, the results from H&E staining indicated that mice subjected to IR‐LND@Lip treatment didn't display noticeable histopathological damage in major organs (Figure 4H). In summary, IR‐LND@Lip demonstrated ideal biocompatibility and held potential for clinical translation.

### 3.5 IR‐LND@Lip Enhanced the Effectiveness of Radio‐Immunotherapy In Vivo

Considering the multifaceted effects of IR‐LND@Lip, including enhanced cytotoxicity of T cells, downregulation of PD‐L1/TGF‐β dual immune proteins, and reversal of the fibrotic tumor microenvironment in vitro, we hypothesized that it can proficiently alleviate T‐cell exhaustion, consequently enhancing the efficacy of radio‐immunotherapy in vivo. As expected, quantitative flow cytometry analysis revealed a significant increase in the infiltration of CD3^+^, CD4^+^, and CD8^+^ T cells at the 4T1 tumor site due to IR‐LND@Lip treatment (Figures 5A–D). What was particularly noteworthy was the remarkable synergistic enhancement observed when combining IR‐LND@Lip with RT. In the IR‐LND@Lip combined with the RT group, the ratios of CD3^+^CD4^+^ T cells and CD3^+^CD8^+^ T cells were 9.64 ± 2.69 times and 12.26 ± 1.70 times, respectively, compared to the control group (Figure 5A–D). Subsequently, tumor tissue sections from each group were subjected to immunofluorescent staining for γ‐H_2_AX, TUNEL, and Ki67, as well as H&E staining, to assess DNA damage, apoptosis, proliferation, and tumor necrosis, respectively, following different treatments. As depicted in Figure 5E, IR‐LND@Lip triggered a specific degree of DNA damage and apoptosis in 4T1 tumor cells, while also restraining tumor cell proliferation to a certain extent. Nonetheless, the IR‐LND@Lip combined with the RT group induced significantly more severe DNA damage and the most pronounced apoptosis of 4T1 tumor cells compared to all other groups. Moreover, the proliferation of 4T1 cells in the IR‐LND@Lip combined with the RT group was significantly inhibited (Figure 5E). Additionally, H&E staining of the tumor indicated that the IR‐LND@Lip combined with the RT group caused the greatest extent of tumor necrosis compared to any other group (Figure S23, Supporting Information). Collectively, these findings strongly suggested that IR‐LND@Lip efficiently enhanced the efficacy of radio‐immunotherapy in vivo.

### 3.6 The Combination Therapy of IR‐LND@Lip and RT Markedly Impeded Primary Tumor Growth and Suppressed Tumor Metastasis In Vivo

Motivated by the exceptional anticancer activity of IR‐LND@Lip in vitro and its remarkable ability to rejuvenate immunity in vivo, we established a 4T1 tumor model to comprehensively assess the synergistic therapeutic effects of IR‐LND@Lip and RT. The treatment protocol was illustrated in Figure 6A, primary tumors were harvested after a 14‐day treatment period. Immunoblotting results of tumor tissues from different treatment groups confirmed that IR‐LND@Lip effectively downregulated the expression of PD‐L1/TGF‐β dual immune proteins, concurrently leading to a reduction in the expression of the fibrotic marker COL1A1 (Figure 6B; Figure S24, Supporting Information). Significantly, IR‐LND@Lip proficiently reversed the RT‐induced upregulation of PD‐L1/TGF‐β proteins and counteracted the fibrotic tumor microenvironment (Figure 6B; Figure S24, Supporting Information), providing further confirmation of its synergistic effect in enhancing radio‐immunotherapy. Concurrently, the growth status of primary 4T1 tumors indicated that the combination of IR‐LND@Lip with RT could maximally inhibit the growth of 4T1 tumors (Figure 6C,D; Figure S25, Supporting Information). Additionally, treatment with IR‐LND@Lip alone demonstrated noteworthy tumor suppression, whereas LND@Lip alone, without combination therapy with RT, didn't appear to achieve a comparable effect (Figure 6C,D; Figure S25, Supporting Information). The final weight results of the collected primary tumors further corroborated these findings (Figure 6E). Moreover, the administration of IR‐LND@Lip nanoadjuvants didn't induce a significant change in the body weight of 4T1 tumor‐bearing mice compared to the control group (Figure 6F), underscoring the favorable biocompatibility of IR‐LND@Lip in vivo.

Furthermore, to explore the impact of IR‐LND@Lip on lung metastasis of 4T1 tumors, the mice were sutured after the removal of the primary 4T1 tumor (on day 14) and continued to be fed for 16 days without any additional treatment to simulate postoperative lung metastasis (Figure 6A). As anticipated, the group treated with the combination of IR‐LND@Lip and RT demonstrated the most effective inhibition of lung metastasis (Figure 6G–J). Compared to the control group, the IR‐LND@Lip combined with the RT group showed a 2.03‐fold reduction in lung weight and a 17.69‐fold decrease in the number of lung metastatic nodules (Figure 6G–J). Additionally, IR‐LND@Lip without RT also exhibited a potent anti‐tumor metastasis effect. However, standalone RT, LND@Lip, and their combination didn't demonstrate efficacy against tumor metastasis (Figure 6G–J). These results suggested that IR‐LND@Lip can synergistically interact with RT to more effectively suppress primary tumor growth and exert a potent inhibitory effect on lung metastasis.

## 4 Discussion

Collagen has been proven to be associated with the failure and poor efficiency of various tumor therapies. Taking RT, for example, apart from lowering the efficacy of RT in inhibiting local tumor growth by impairing T cell infiltration and limiting oxygen perfusion, radiation‐induced pulmonary fibrosis (RIPF) often occurred (50%) when facing the enhanced collagen and fibronectin after the activation of lung fibroblasts by RT.^[^ ^19^ ^]^ When the malignant irreversible RIPF was induced, the normal function of lung tissues usually was impaired, which then could even lead to the death of tumor patients.^[^ ^19^, ^20^ ^]^ However, at present, few methods have been discovered to effectively cure or remiss RIPF effectively.^[^ ^19^, ^21^ ^]^ In this study, we revealed that IR‐LND@Lip mediated OXPHOS inhibition could effectively reverse T cell exhaustion by depressing PD‐L1, TGF‐β, and collagen expression, when then obviously inhibited local 4T1 tumor growth and its lung metastasis by enhancing CD3^+^, CD4^+^, and CD8^+^ T cell infiltration, as well as generating enough DNA damage to cause tumor cell apoptosis (Figures 2, 3, 4, 5, 6). As recently proved, heptamethine cyanine dye like IR780 could selectively be accumulated at the RIPF site due to the enhanced expression of SLCO2A1 in RIPF.^[^ ^22^ ^]^ Considering the fact that IR‐LND also was a typical heptamethine cyanine dye, IR‐LND@Lip with collagen depletion capacity may also possess RIPF‐targeting capacity to more effectively slow or remiss the development of RIPF.^[^ ^22^ ^]^ But, such speculation still needs to be proved in our further research. All in all, IR‐LND@Lip may work as a multi‐functional immune‐regulation nanosystem to sensitize RT, as well as remissing RIPF development.

At present, the aberrant high tumor interstitial pressure partly induced by collagen could cause tumor hypoxia, impair drug accumulation, and induce T cell exhaustion to impair the therapeutic effect of tumor therapies including but not only RT.^[^ ^23^ ^]^ Similarly to RT, collagen‐caused high tumor interstitial pressure could also induce the acquired resistance to photodynamic therapy (PDT), chemotherapy, sonodynamic therapy (SDT), adoptive cell therapy, et al. since the efficacy of these tumor therapies all depend on the immune status of tumors, among which the amounts of T cells that infiltrated in tumors was important.^[^ ^24^ ^]^ Besides, due to the hypoxia tumor status caused by collagen‐impaired oxygen perfusion, amounts of the generated reactive oxygen species by PDT or SDT were limited.^[^ ^23^, ^25^ ^]^ Moreover, the drug accumulation behavior of SDT, PDT, or chemotherapy drugs was always limited since the high tumor interstitial pressure impaired this process, which then lowered the tumor cell‐killing efficacy of these therapies.^[^ ^26^ ^]^ In this study, we revealed that IR‐LND@Lip could affect collagen expression in tumors to enhance T cell infiltration and reverse tumor hypoxia by decreasing oxygen consumption and enhancing oxygen perfusion (Figures 2, 3, 4, 5, 6). Thus, IR‐LND@Lip may also be used as a multi‐functional adjuvant to sensitize PDT, SDT, and chemotherapy by reversing tumor hypoxia, enhancing drug accumulation, and amplifying T cell infiltration.

Recently, some studies have shown that the AMPK pathway plays a role in influencing the metastasis process of breast cancers.^[^ ^27^ ^]^ Consequently, IR‐LND might not solely impact tumor metastasis through collagen inhibition. This represents a promising avenue for further research. In addition, overwhelming evidence revealed that AMPK pathway activation played a vital role in the prevention, treatment, and healing of some chronic diseases such as idiopathic fibrosis, obesity, aging, non‐alcoholic fatty liver disease, fungal‐related disease, type 2 diabetes, and so on.^[^ ^28^ ^]^ In this study, it was revealed that IR‐LND@Lip could effectively induce AMPK pathway activation with desirable biosecurity in a relatively low dosage (Figure 4). Thus, IR‐LND@Lip could expand the clinical application field of OXPHOS inhibitors or nanosystems, further supporting its clinical translation not only in various tumor therapy sensitizations but also in some chronic disease treatments

Overall, IR‐LND@Lip more effectively sensitizes RT by generating more DNA damage and transforming cold tumors into hot ones through hypoxia reversion and immune activation by PD‐L1, collagen, and TGF‐β co‐inhibition. This multifunctional nanoadjuvant shows potential as a novel, simple, and efficient strategy for regulating OXPHOS processes, making it applicable to the treatment of various clinical diseases.

## 5 Conclusion

In this study, we revealed that OXPHOS inhibition mediated by LND could effectively, easily, and simultaneously inhibit PD‐L1 expression, decrease collagen secretion, and depress TGF‐β secretion in vitro via regulating mitochondria energy metabolism. Then, to solve the defects of high‐dosage and non‐specific targeting capacity of LND, IR‐LND was prepared by combining the mitochondria‐targeted heptamethine cyanine dye IR‐68 with LND and then loaded with liposomes (Lip) to create IR‐LND@Lip nanoparticles, which was about 100‐fold potent than free LND (300 µm LND vs 2 µm IR‐LND) in depressing TGF‐β and collagen expression. Following this, IR‐LND@Lip nanoadjuvants effectively sensitized RT in vitro through the generation of more DNA damage by decreasing oxygen consumption and impairing the DNA damage repair process partly by depressing PD‐L1 expression. Moreover, the T cell infiltrations of CD3^+^, CD4^+^, and CD8^+^ T cells, as well as their anti‐tumor capacity, were amplified via immune‐suppression microenvironment reversion through high‐effective collagen, PD‐L1, and TGF‐β co‐inhibition by IR‐LND@Lip in vivo. These two aspects mediated by IR‐LND@Lip altogether more effectively sensitized RT in 4T1 breast tumors by inducing more tumor cell apoptosis and reversing immune tolerance with almost totally inhibited local 4T1 tumor growth and depressed lung metastasis. All in all, this simple but effective method could expand the clinical application field of mitochondrial metabolism disruption agents in tumor combination therapy, especially in tumor radiotherapy therapy sensitization.

## Conflict of Interest

The authors declare no conflict of interest.

## Author Contributions

Z.Z., C.L., and C.L. contributed equally to this work. W.X., Z.Z., L.W., C.X., and J.S. conceived the project. Z.Z., C.L., C.L., S.T., L.Z., and W.H. performed the experiments and analyzed the results. Z.Z., W.X., C.L., and J.S. wrote the manuscript. Z.Z., J.S., W.X., L.W., C.L., C.X., and C.L. discussed the results and reviewed the manuscript.

## Supporting information

Supporting Information

## Data Availability

The data that support the findings of this study are available in the supplementary material of this article.

## References

[1] a) D. H. Peng , B. L. Rodriguez , L. Diao , L. Chen , J. Wang , L. A. Byers , Y. Wei , H. A. Chapman , M. Yamauchi , C. Behrens , G. Raso , L. M. S. Soto , E. R. P. Cuentes , I. I. Wistuba , J. M. Kurie , D. L. Gibbons , Nat. Commun. 2020, 11, 4520; 32908154 10.1038/s41467-020-18298-8 PMC7481212

[2] a) J. Li , L. Xie , W. Sang , W. Li , G. Wang , J. Yan , Z. Zhang , H. Tian , Q. Fan , Y. Dai , Angew. Chem. Int. Ed. Engl. 2022, 61, e202200830; 35174599 10.1002/anie.202200830

[3] a) X. Bao , L. Xie , J. Exp. Clin. Cancer Res. 2022, 41, 222; 35836249 10.1186/s13046-022-02430-1 PMC9284706

[4] a) L. Tanner , A. B. Single , R. K. V. Bhongir , M. Heusel , T. Mohanty , C. A. Q. Karlsson , L. Pan , C. M. Clausson , J. Bergwik , K. Wang , C. K. Andersson , R. M. Oommen , J. S. Erjefalt , J. Malmstrom , O. Wallner , I. Boldogh , T. Helleday , C. Kalderen , A. Egesten , Nat. Commun. 2023, 14, 643; 36746968 10.1038/s41467-023-36314-5 PMC9902543

[5] a) Z. Zhou , H. Wang , J. Li , X. Jiang , Z. Li , J. Shen , Int. J. Biol. Macromol. 2023, 254, 127911; 37939766 10.1016/j.ijbiomac.2023.127911

[6] a) T. A. Yap , N. Daver , M. Mahendra , J. Zhang , C. Kamiya‐Matsuoka , F. Meric‐Bernstam , H. M. Kantarjian , F. Ravandi , M. E. Collins , M. E. D. Francesco , E. E. Dumbrava , S. Fu , S. Gao , J. P. Gay , S. Gera , J. Han , D. S. Hong , E. J. Jabbour , Z. Ju , D. D. Karp , A. Lodi , J. R. Molina , N. Baran , A. Naing , M. Ohanian , S. Pant , N. Pemmaraju , P. Bose , S. A. Piha‐Paul , J. Rodon , et al., Nat. Med. 2023, 29, 115; 36658425 10.1038/s41591-022-02103-8 PMC11975418

[7] a) J. Chen , Y. Zhu , C. Wu , J. Shi , Chem. Soc. Rev. 2023, 52, 973; 36597879 10.1039/d2cs00479h

[8] a) Z. Zhou , N. Jiang , J. Chen , C. Zheng , Y. Guo , R. Ye , R. Qi , J. Shen , J. Nanobiotechnol. 2021, 19, 375; 10.1186/s12951-021-01124-8 PMC8600872 34794446

[9] a) J. H. Cha , W. H. Yang , W. Xia , Y. Wei , L. C. Chan , S. O. Lim , C. W. Li , T. Kim , S. S. Chang , H. H. Lee , J. L. Hsu , H. L. Wang , C. W. Kuo , W. C. Chang , S. Hadad , C. A. Purdie , A. M. McCoy , S. Cai , Y. Tu , J. K. Litton , E. A. Mittendorf , S. L. Moulder , W. F. Symmans , A. M. Thompson , H. Piwnica‐Worms , C. H. Chen , K. H. Khoo , M. C. Hung , Mol. Cell 2018, 71, 606; 30118680 10.1016/j.molcel.2018.07.030 PMC6786495

[10] a) A. Costa , Y. Kieffer , A. Scholer‐Dahirel , F. Pelon , B. Bourachot , M. Cardon , P. Sirven , I. Magagna , L. Fuhrmann , C. Bernard , C. Bonneau , M. Kondratova , I. Kuperstein , A. Zinovyev , A. M. Givel , M. C. Parrini , V. Soumelis , A. Vincent‐Salomon , F. Mechta‐Grigoriou , Cancer Cell 2018, 33, 463; 29455927 10.1016/j.ccell.2018.01.011

[11] H. Han , Y. Hou , X. Chen , P. Zhang , M. Kang , Q. Jin , J. Ji , M. Gao , J. Am. Chem. Soc. 2020, 142, 4944. 32069041 10.1021/jacs.0c00650

[12] a) N. A. Daurio , S. W. Tuttle , A. J. Worth , E. Y. Song , J. M. Davis , N. W. Snyder , I. A. Blair , C. Koumenis , Cancer Res. 2016, 76, 3295; 27020861 10.1158/0008-5472.CAN-15-2197 PMC4895922

[13] a) G. Cheng , Q. Zhang , J. Pan , Y. Lee , O. Ouari , M. Hardy , M. Zielonka , C. R. Myers , J. Zielonka , K. Weh , A. C. Chang , G. Chen , L. Kresty , B. Kalyanaraman , M. You , Nat. Commun. 2019, 10, 2205; 31101821 10.1038/s41467-019-10042-1 PMC6525201

[14] a) J. Chen , F. Ren , W. Cao , Z. Wu , G. Ju , C. Xiao , W. Wu , S. Gao , C. Xu , Y. Gao , Nanomedicine 2021, 34, 102370; 33713859 10.1016/j.nano.2021.102370

[15] Y. Liu , Z. Zhou , J. Hou , W. Xiong , H. Kim , J. Chen , C. Zheng , X. Jiang , J. Yoon , J. Shen , Adv. Mater. 2022, 34, 2206121. 10.1002/adma.202206121 36017886

[16] J. Chen , Z. Zhou , C. Zheng , Y. Liu , R. Hao , X. Ji , Q. Xi , J. Shen , Z. Li , Carbohydr. Polym. 2022, 277, 118869. 34893274 10.1016/j.carbpol.2021.118869

[17] a) Z. Zhou , C. Zheng , Y. Liu , W. Luo , H. Deng , J. Shen , Carbohydr. Polym. 2022, 295, 119878; 35989018 10.1016/j.carbpol.2022.119878

[18] a) M. Li , X. Jin , T. Liu , F. Fan , F. Gao , S. Chai , L. Yang , Nat. Commun. 2022, 13, 4137; 35842431 10.1038/s41467-022-31882-4 PMC9288426

[19] a) D. S. Glass , D. Grossfeld , H. A. Renna , P. Agarwala , P. Spiegler , J. DeLeon , A. B. Reiss , Clin. Respir. J. 2022, 16, 84; 35001525 10.1111/crj.13466 PMC9060042

[20] J. Meng , Y. Li , C. Wan , Y. Sun , X. Dai , J. Huang , Y. Hu , Y. Gao , B. Wu , Z. Zhang , K. Jiang , S. Xu , J. F. Lovell , Y. Hu , G. Wu , H. Jin , K. Yang , JCI Insight 2021, 6, e146334. 34877934 10.1172/jci.insight.146334 PMC8675198

[21] A. Mukherjee , M. W. Epperly , R. Fisher , W. Hou , D. Shields , M. Saiful Huq , P. M. Pifer , R. Mulherkar , T. J. Wilhite , H. Wang , P. Wipf , J. S. Greenberger , Cell Death Discov. 2023, 9, 252. 37460469 10.1038/s41420-023-01538-3 PMC10352363

[22] a) Z. Chen , Z. Wang , T. Jin , G. Shen , Y. Wang , X. Tan , Y. Gan , F. Yang , Y. Liu , C. Huang , Y. Zhang , X. Fu , C. Shi , Theranostics 2019, 9, 6797; 31660069 10.7150/thno.36375 PMC6815952

[23] a) Z. Zhou , B. Zhang , S. Wang , W. Zai , A. Yuan , Y. Hu , J. Wu , Small 2018, 14, e1801694; 30307696 10.1002/smll.201801694

[24] X. Li , T. Yong , Z. Wei , N. Bie , X. Zhang , G. Zhan , J. Li , J. Qin , J. Yu , B. Zhang , L. Gan , X. Yang , Nat. Commun. 2022, 13, 2794. 35589680 10.1038/s41467-022-30306-7 PMC9120472

[25] A. Martinez‐Ordonez , A. Duran , M. Ruiz‐Martinez , T. Cid‐Diaz , X. Zhang , Q. Han , H. Kinoshita , Y. Muta , J. F. Linares , H. Kasashima , Y. Nakanishi , M. Omar , S. Nishimura , L. Avila , M. Yashiro , K. Maeda , T. Pannellini , A. Pigazzi , G. Inghirami , L. Marchionni , D. Sigal , M. T. Diaz‐Meco , J. Moscat , Cancer Cell 2023, 41, 252. 36525970 10.1016/j.ccell.2022.11.016 PMC9931663

[26] a) G. Caligiuri , D. A. Tuveson , Cancer Cell 2023, 41, 434; 36917949 10.1016/j.ccell.2023.02.015 PMC11022589

[27] Y. Yi , D. Chen , J. Ao , W. Zhang , J. Yi , X. Ren , J. Fei , F. Li , M. Niu , H. Chen , Y. Luo , Z. Luo , Z. J. Xiao , Proc. Natl. Acad. Sci. USA 2020, 117, 8013. 32193335 10.1073/pnas.1914786117 PMC7148563

[28] a) H. Lee , F. Zandkarimi , Y. Zhang , J. K. Meena , J. Kim , L. Zhuang , S. Tyagi , L. Ma , T. F. Westbrook , G. R. Steinberg , D. Nakada , B. R. Stockwell , B. Gan , Nat. Cell Biol. 2020, 22, 225; 32029897 10.1038/s41556-020-0461-8 PMC7008777

