# Supplementary material for: Mitochondria‐Targeted Nanoadjuvants Induced Multi‐Functional Immune‐Microenvironment Remodeling to Sensitize Tumor Radio‐Immunotherapy
Source: Adv Sci (Weinh). 2024 May 5;11(26):2400297. doi: 10.1002/advs.202400297 (PMC11234464; doi:10.1002/advs.202400297)
Supplement: Supplementary file 1 — Supporting Information [file ADVS-11-2400297-s001.pdf]

ADVANCED SCIENCE
Open Access

Supporting Information

for *Adv. Sci.*, DOI 10.1002/advs.202400297

Mitochondria-Targeted Nanoadjuvants Induced Multi-Functional Immune-Microenvironment Remodeling to Sensitize Tumor Radio-Immunotherapy

Zaigang Zhou, Cheng Li, Chao Li, Lei Zhou, Shuo Tan, Weibin Hou, Congying Xie, Long Wang, Jianliang Shen* and Wei Xiong**

Mitochondria-targeted Nanoadjuvants Induced Multi-functional Immune-microenvironment Remodeling to Sensitize Tumor Radio-immunotherapy

Zaigang Zhou[2,#], Cheng Li[1,#], Chao Li[1,#], Lei Zhou[1], Shuo Tan[1], Weibin Hou[1], Congying Xie[4], Long Wang[1,*], Jianliang Shen[2,3,*], Wei Xiong[1,*]

[1] Department of Urology, The Third Xiangya Hospital of Central South University, Changsha, 410013, China.

[2] National Engineering Research Center of Ophthalmology and Optometry, Eye Hospital, Wenzhou Medical University, Wenzhou, Zhejiang 325027, China.

[3] Zhejiang Engineering Research Center for Tissue Repair Materials, Wenzhou Institute, University of Chinese Academy of Sciences, Wenzhou, Zhejiang 325001, China.

[4] Department of the Second Affiliated Hospital of Wenzhou Medical University, Wenzhou, 325000, China.

[#] These authors contributed equally to this paper.

[*] **Corresponding to:**

xiongweixymn@163.com (Wei Xiong), sjl1@wmu.edu.cn, shenjl@wiucas.ac.cn (Jianliang Shen), wanglong@csu.edu.cn (Long Wang)

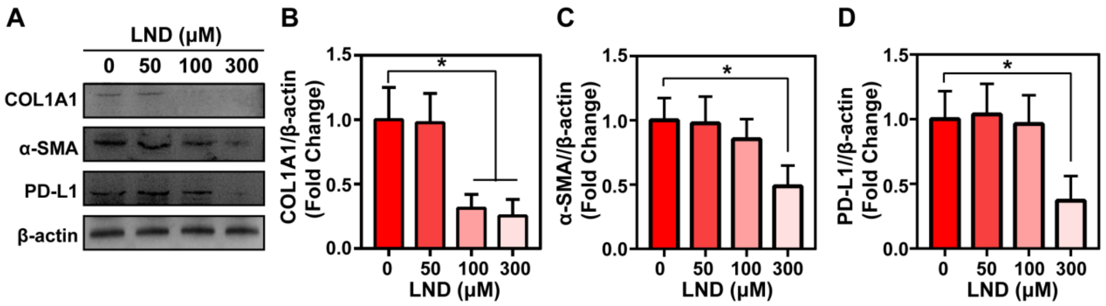

Figure S1. (A) Detection of the expression of COL1A1, α-SMA, and PD-L1 protein in MB49 cells by western blotting after treatment with indicated doses of LND (n = 3). (B-D) The quantitative analysis of the expression of COL1A1, α-SMA, and PD-L1 protein was performed by ImageJ. Data were demonstrated as mean ± SD. Statistical analysis was performed via the two-tailed Student's *t*-test. * $p < 0.05$.

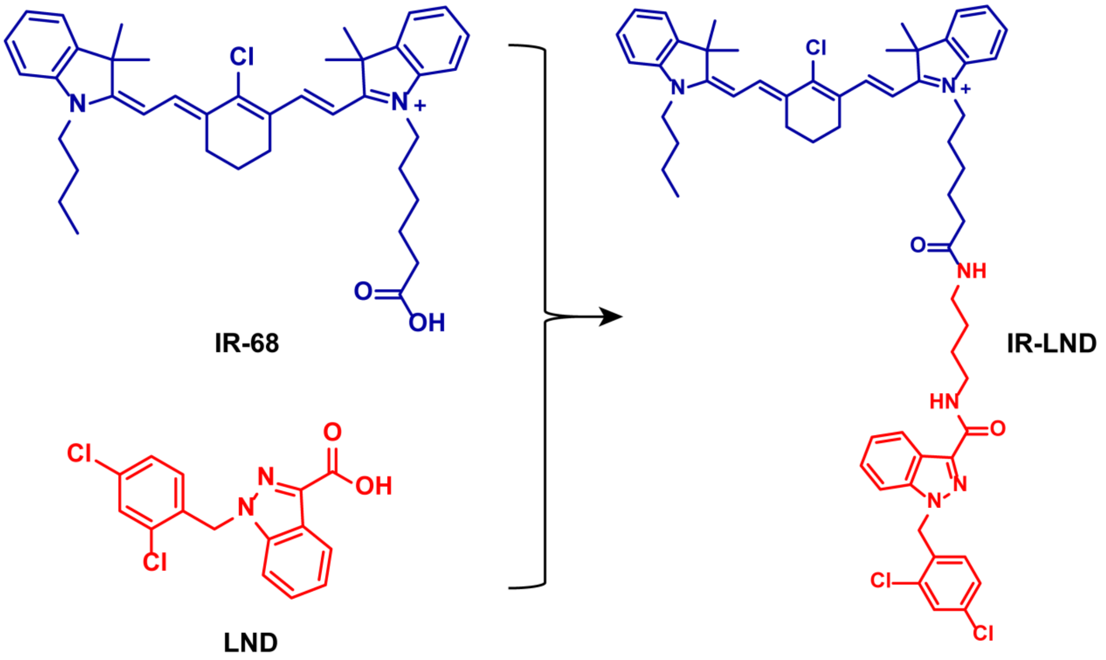

Figure S2. Synthesis of IR-LND

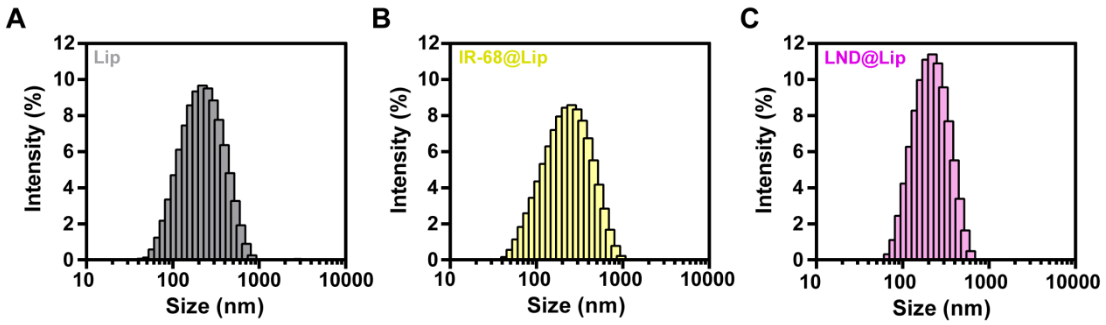

Figure S3. (A-C) Hydrodynamic diameters of Blank Lip, IR-68@Lip, and LND@Lip nanoparticles.

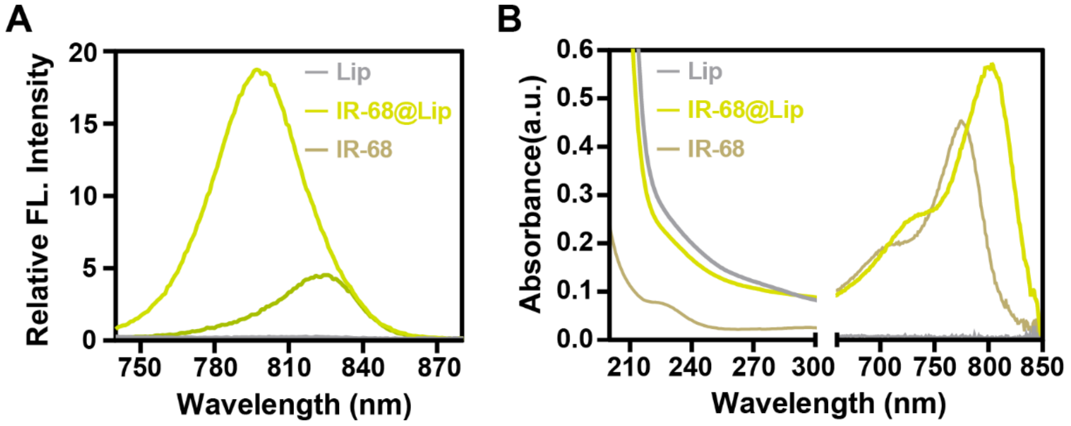

Figure S4. (A) Fluorescence spectra of Blank Lip, IR-68, and IR-68@Lip in deionized water. (B) UV-VIS spectra of Blank Lip, IR-68, and IR-68@Lip in deionized water.

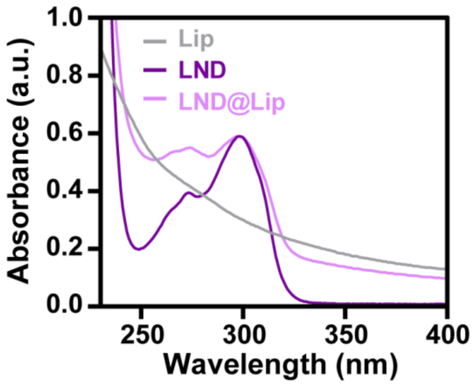

Figure S5. UV-VIS spectra of Blank Lip, LND, and LND@Lip in deionized water.

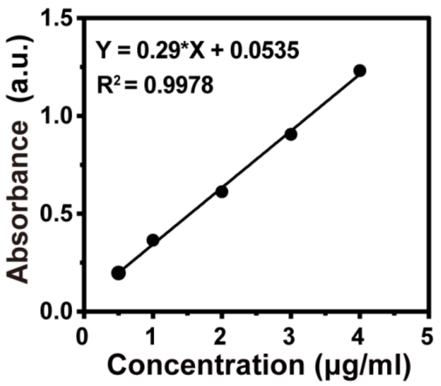

Figure S6. The standard curve of IR-LND was examined by UV–vis spectrophotometry.

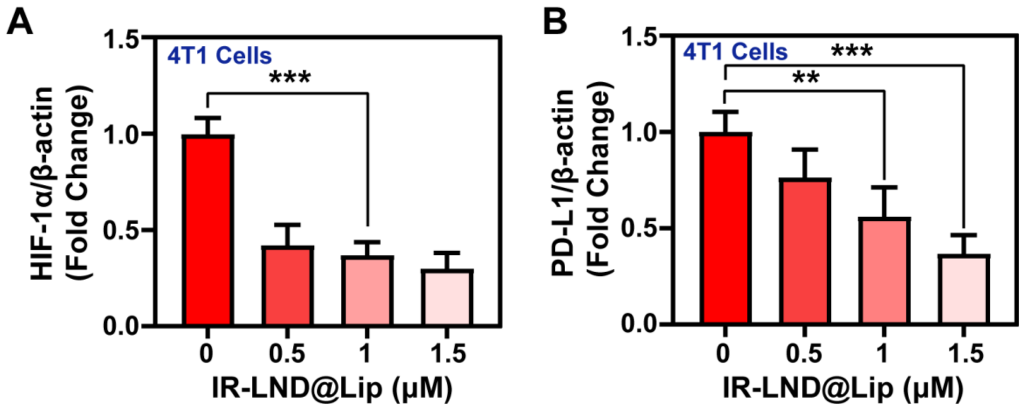

Figure S7. (A-B)The quantitative analysis of the expression of HIF-1α or PD-L1 protein was performed by ImageJ (n = 3). Data were demonstrated as mean ± SD. Statistical analysis was performed via the two-tailed Student's *t*-test. ** $p < 0.01$, *** $p < 0.001$.

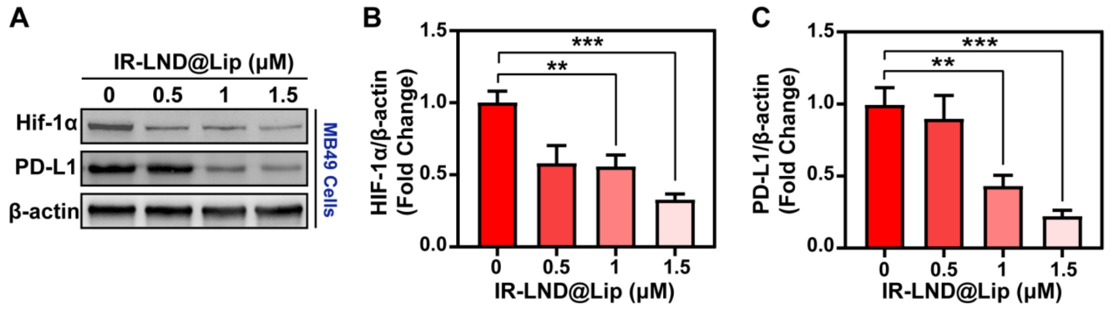

Figure S8. (A) Detection of the expression of HIF-1α and PD-L1 protein in MB49 cells by western blotting after treatment with indicated doses of IR-LND@Lip (n = 3). (B-C) The quantitative analysis of the expression of HIF-1α or PD-L1 protein was performed by ImageJ. Data were demonstrated as mean ± SD. Statistical analysis was performed via the two-tailed Student's *t*-test. ** $p < 0.01$, *** $p < 0.001$.

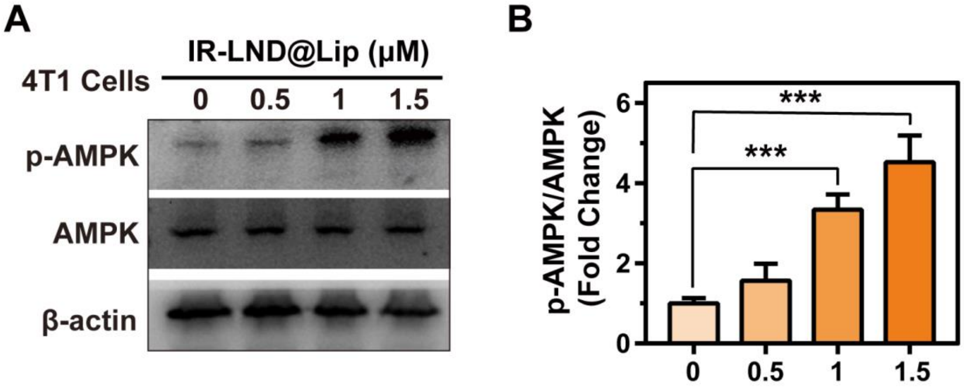

Figure S9. (A) Detection of the expression of p-AMPK and AMPK protein in 4T1 cells by western blotting after treatment with indicated doses of IR-LND@Lip (n = 3). (B) The quantitative analysis of the expression of p-AMPK/AMPK was performed by ImageJ. Data were demonstrated as mean ± SD. Statistical analysis was performed via the two-tailed Student's t-test. *** $p < 0.001$.

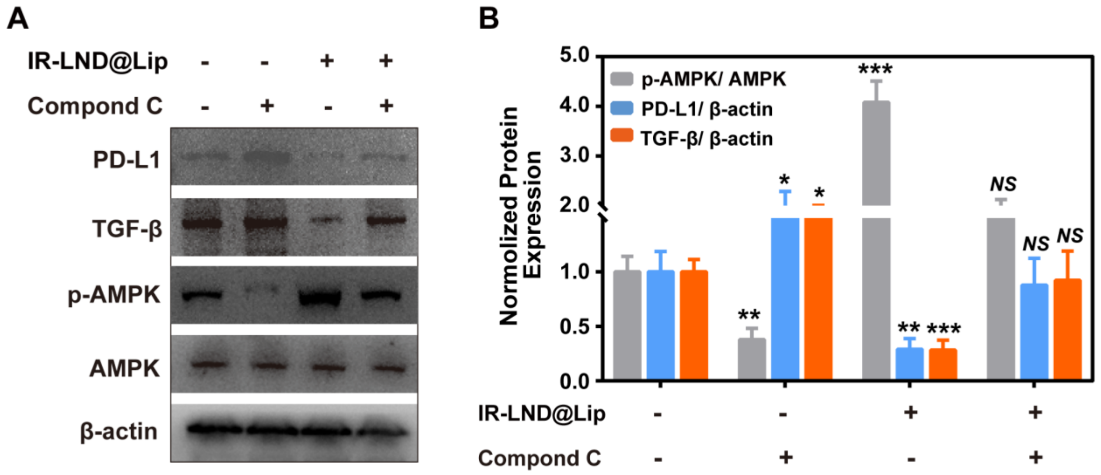

Figure S10. (A-B) The expression of PD-L1, TGF-β, p-AMPK, and AMPK protein after IR-LND@Lip treatment with or without the addition of Compound C (10 μM) and corresponding quantitative analysis by ImageJ (n = 3). Data were demonstrated as mean ± SD. Statistical analysis was performed via the two-tailed Student's t-test. * $p < 0.05$, ** $p < 0.01$, and *** $p < 0.001$. *NS* indicates no statistically significant difference ($p > 0.05$).

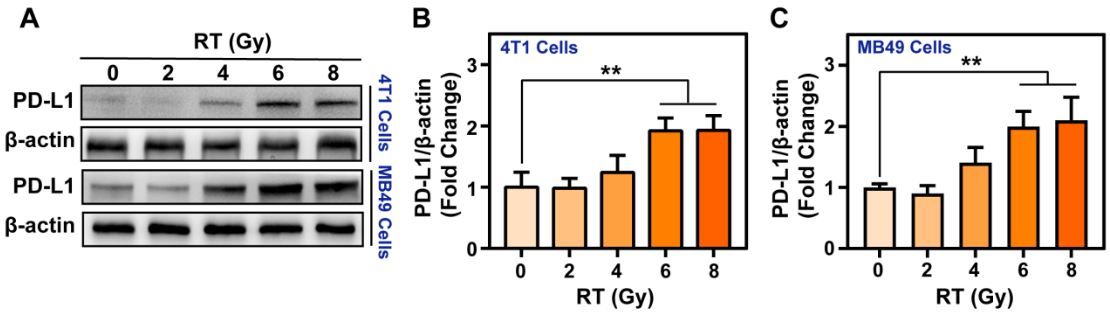

Figure S11. (A) Detection of the expression of PD-L1 protein in 4T1 or MB49 cells by western blotting after treatment with different radiation doses (n = 3). (B-C) The quantitative analysis of the expression of PD-L1 proteins in 4T1 or MB49 cells was performed by ImageJ. Data were demonstrated as mean ± SD. Statistical analysis was performed via the two-tailed Student's *t*-test. ** $p < 0.01$.

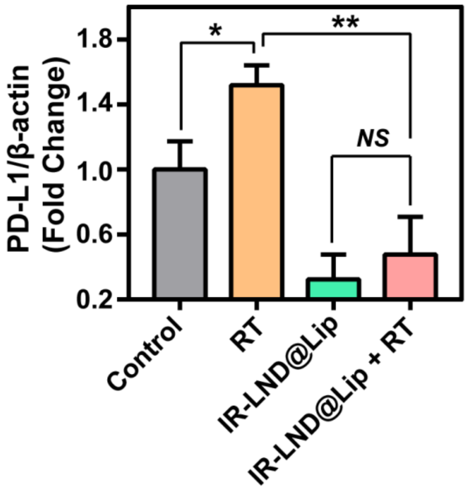

Figure S12. The quantitative analysis of the expression of PD-L1 proteins in 4T1 cells after different treatments was performed by ImageJ (n = 3). Data were demonstrated as mean ± SD. Statistical analysis was performed via the two-tailed Student's *t*-test. * $p < 0.05$, ** $p < 0.01$, *NS* indicates no statistically significant difference ($p > 0.05$).

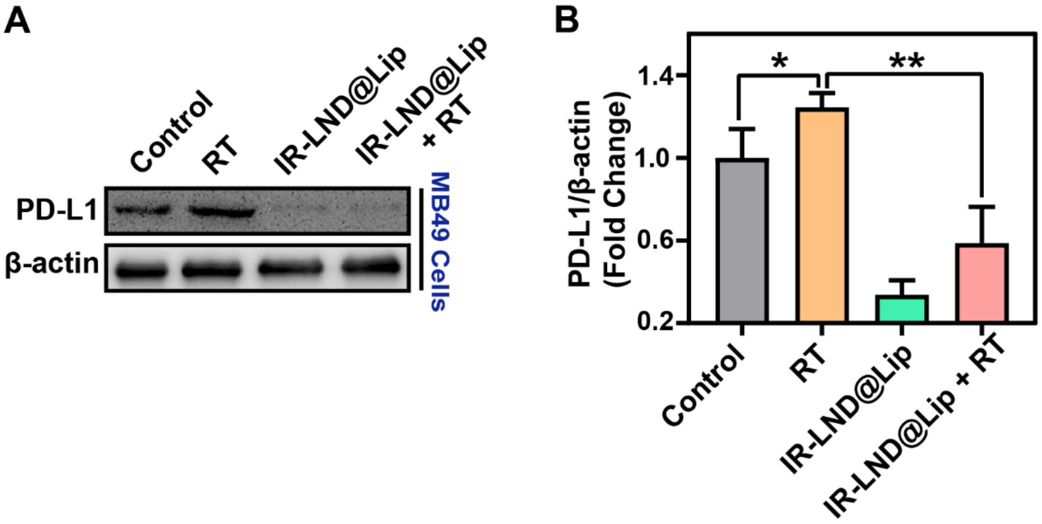

Figure S13. (A) Detection of the expression of PD-L1 protein in MB49 cells by western blotting after different treatments (n = 3). (B) The quantitative analysis of the expression of PD-L1 proteins in MB49 cells after different treatments was performed by ImageJ. Data were demonstrated as mean ± SD. Statistical analysis was performed via the two-tailed Student's *t*-test. * $p < 0.05$, ** $p < 0.01$.

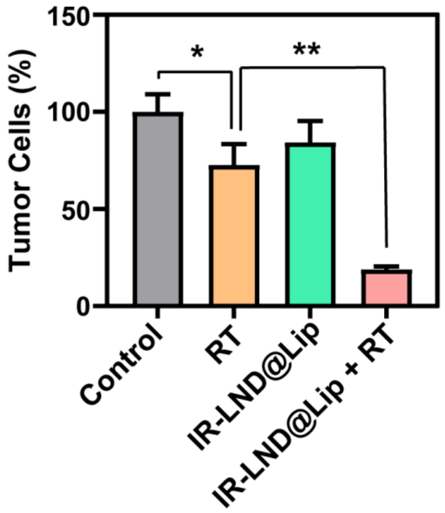

Figure S14. The quantitative analysis of the 4T1 cell clones after different treatments with IR-LND@Lip (1 μM) with or without RT co-treatment (n = 3). Data were demonstrated as mean ± SD. Statistical analysis was performed via the two-tailed Student's *t*-test. * $p < 0.05$, ** $p < 0.01$.

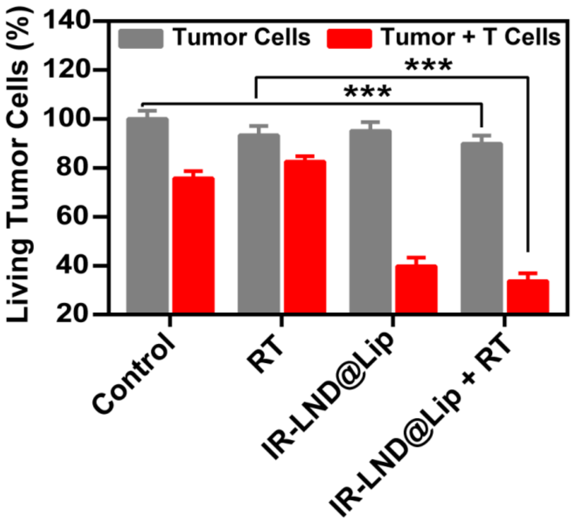

Figure S15. The quantitative analysis of IR-LND@Lip and RT on T cell killing of tumor cells (n = 3). Data were demonstrated as mean ± SD. Statistical analysis was performed via the two-tailed Student's *t*-test. *** $p < 0.001$.

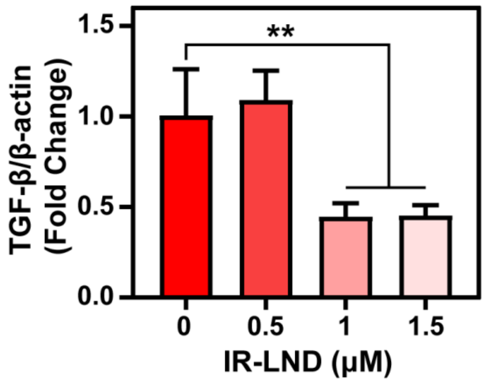

Figure S16. The quantitative analysis of the expression of TGF-β protein in 4T1 cells after treatment with indicated doses of IR-LND was performed by ImageJ (n = 3). Data were demonstrated as mean ± SD. Statistical analysis was performed via the two-tailed Student's t-test. ** $p < 0.01$.

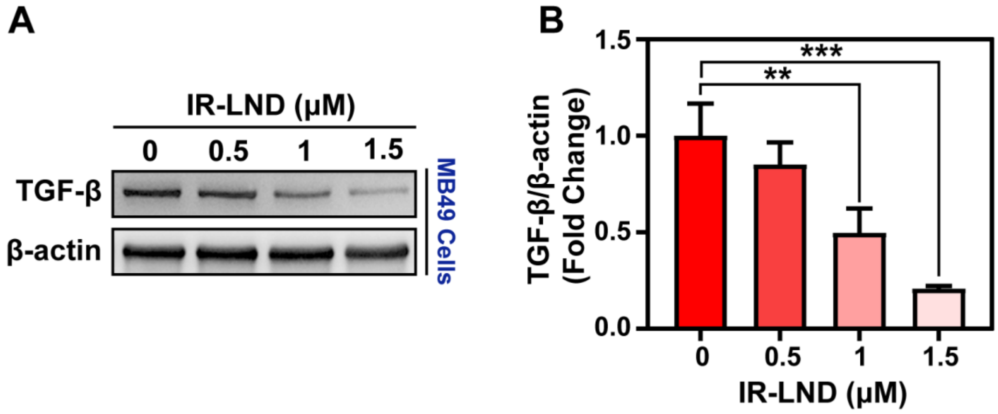

Figure S17. (A) Detection of the expression of TGF-β protein in MB49 cells by western blotting after treatment with indicated doses of IR-LND (n = 3). (B) The quantitative analysis of the expression of TGF-β protein in MB49 cells after treatment with indicated doses of IR-LND was performed by ImageJ. Data were demonstrated as mean ± SD. Statistical analysis was performed via the two-tailed Student's t-test. ** $p < 0.01$, *** $p < 0.001$.

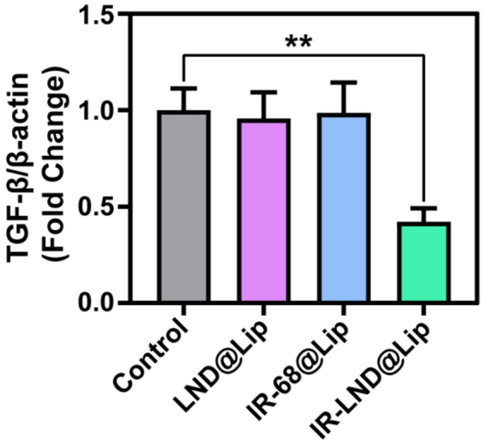

Figure S18. The quantitative analysis of the expression of TGF-β protein in 4T1 cells after different treatments was performed by ImageJ (n = 3). Data were demonstrated as mean ± SD. Statistical analysis was performed via the two-tailed Student's t-test. ** $p < 0.01$.

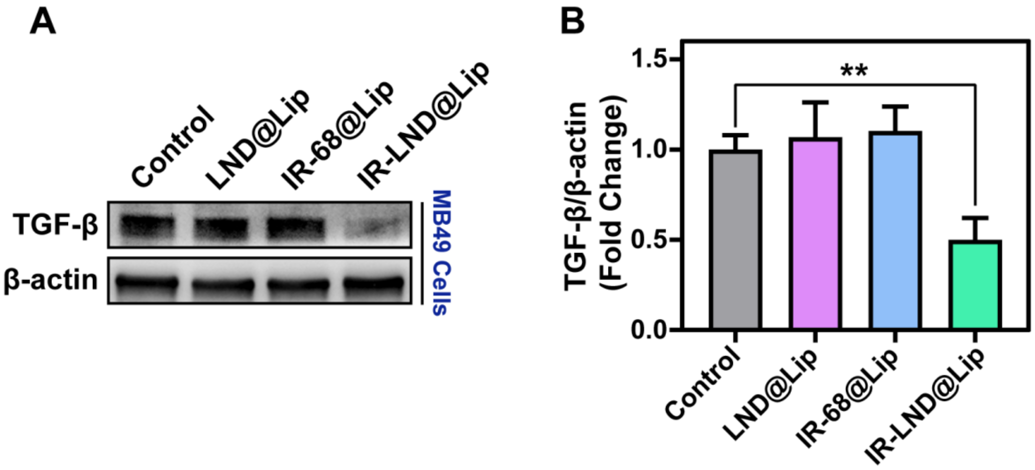

Figure S19. (A) Detection of the expression of TGF-β protein in MB49 cells by western blotting after different treatments (n = 3). (B) The quantitative analysis of the expression of TGF-β protein in MB49 cells after different treatments was performed by ImageJ. Data were demonstrated as mean ± SD. Statistical analysis was performed via the two-tailed Student's t-test. ** $p < 0.01$.

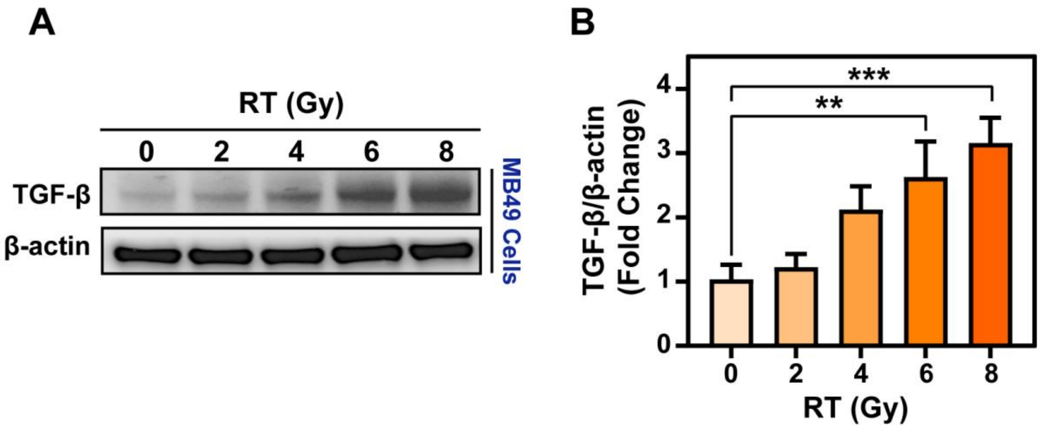

Figure S20. (A-B) Detection of the expression of TGF-β protein in MB49 cells by western blotting after treatment with different radiation doses and further quantification by Image J (n = 3). Data were demonstrated as mean ± SD. Statistical analysis was performed via the two-tailed Student's t-test. ** $p < 0.01$, *** $p < 0.001$.

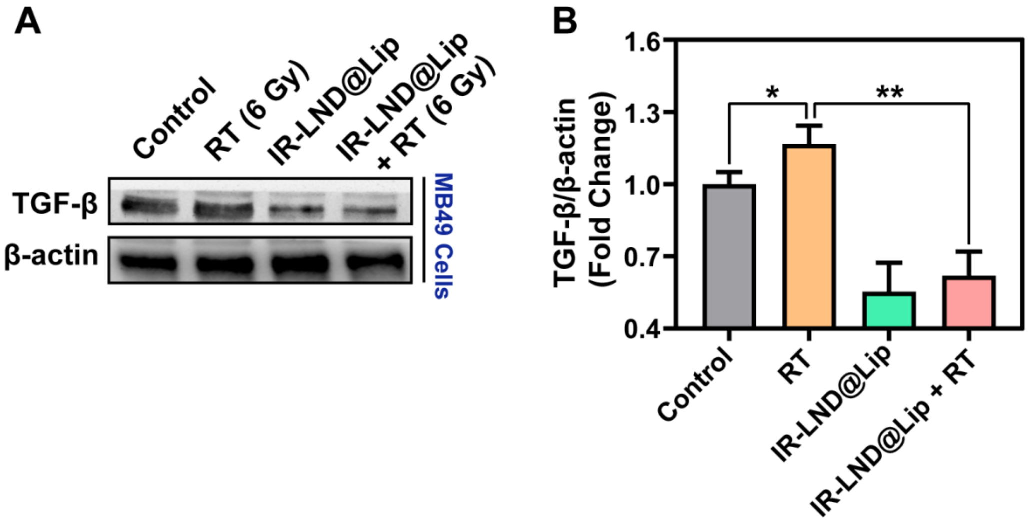

Figure S21. (A-B) Detection of the expression of TGF-β protein in MB49 cells by western blotting after different treatments with indicated doses of IR-LND@Lip (0 or 1 μM) with or without RT co-treatment and further quantification by Image J (n = 3). Data were demonstrated as mean ± SD. Statistical analysis was performed via the two-tailed Student's t-test. * $p < 0.05$, ** $p < 0.01$.

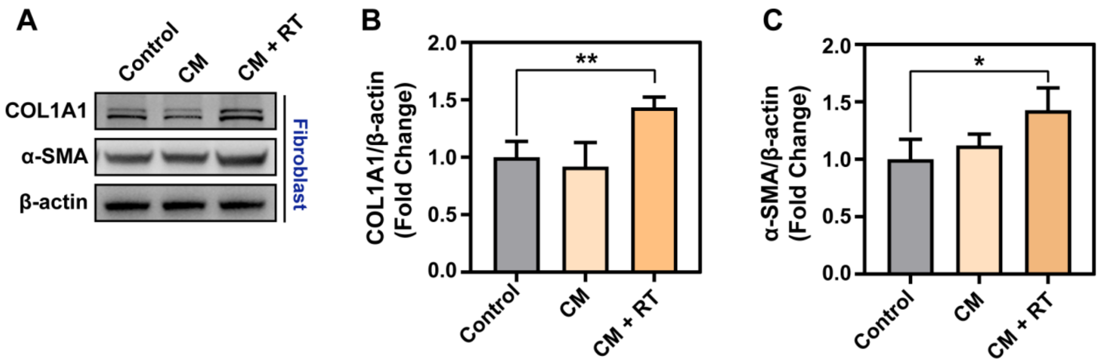

Figure S22. (A-C) Detection of the expression of COL1A1 and α-SMA protein in fibroblasts by western blotting after indicated treatments and further quantification by Image J (n = 3). CM stands for the conditional culture medium (a 1:1 mixture of complete culture medium with 20% FBS and culture medium derived from irradiated 4T1 cells). Data were demonstrated as mean ± SD. Statistical analysis was performed via the two-tailed Student's t-test. * $p < 0.05$, ** $p < 0.01$.

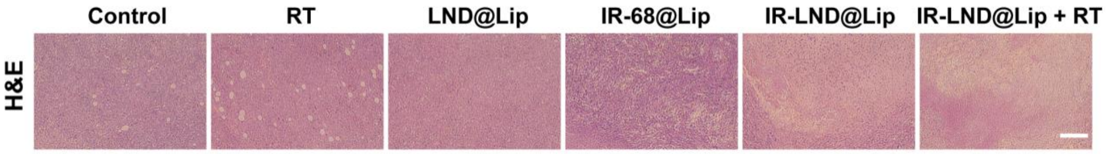

Figure S23. Representative H&E staining images in 4T1 tumors collected from the mice after different treatments, scale bar = 100 μm.

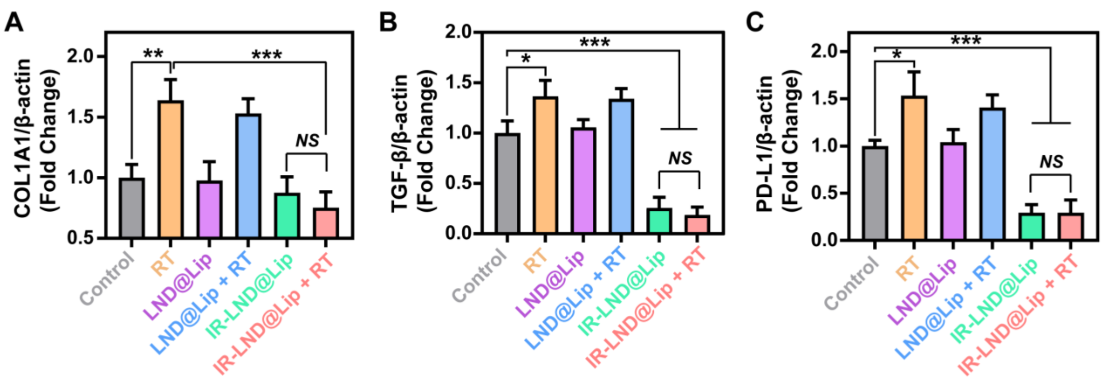

Figure S24. (A-C)The quantitative analysis of the expression of COL1A1, TGF-β, and PD-L1 proteins in 4T1 tumors after different treatments *in vivo* was performed by ImageJ (n = 3). Data were demonstrated as mean ± SD. Statistical analysis was performed via the two-tailed Student's t-test. * $p < 0.05$, ** $p < 0.01$, *** $p < 0.001$, *NS* indicates no statistically significant difference ($p > 0.05$).

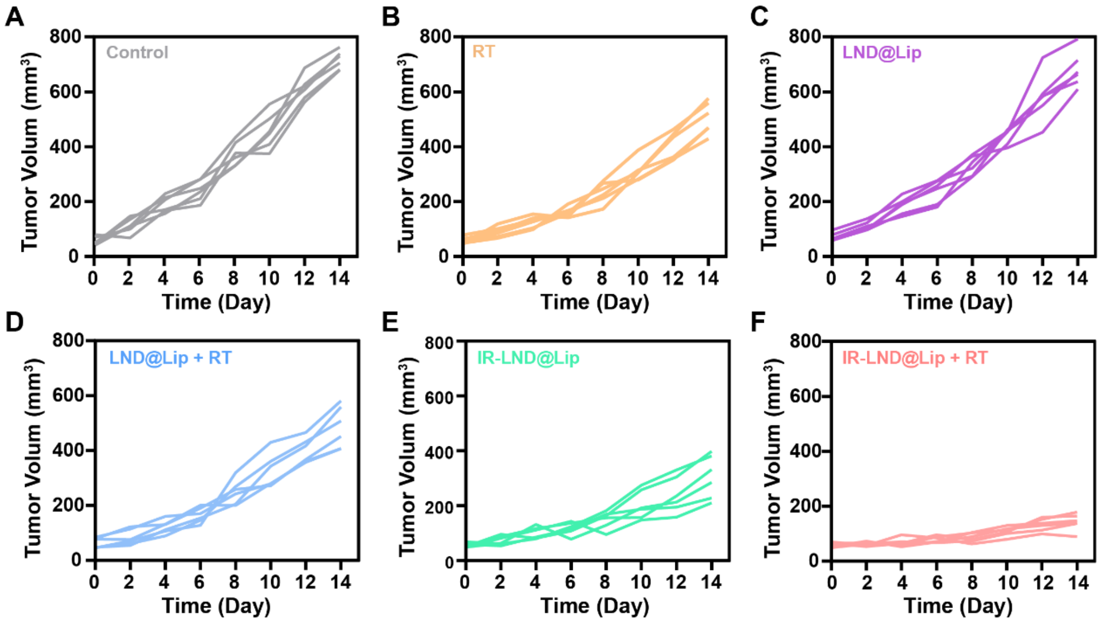

Figure S25. Primary tumor growth curves of each mouse in different treatment groups within 14 days.
